# Spatial and Temporal Protein Modules Signatures Associated with Alzheimer Disease in 3xTg-AD Mice Are Restored by Early Ubiquinol Supplementation

**DOI:** 10.3390/antiox12030747

**Published:** 2023-03-19

**Authors:** Emilio Llanos-González, Francisco J. Sancho-Bielsa, Javier Frontiñán-Rubio, Yoana Rabanal-Ruíz, Sonia García-Carpintero, Eduardo Chicano, Isabel Úbeda-Banon, Alicia Flores-Cuadrado, Lydia Giménez-Llort, Francisco Javier Alcaín, Juan Ramón Peinado, Mario Durán-Prado

**Affiliations:** 1 Department of Medical Sciences, Faculty of Medicine, University of Castilla-La Mancha, 13071 Ciudad Real, Spain; 2 Oxidative Stress and Neurodegeneration Group, Faculty of Medicine, Regional Centre for Biomedical Research, University of Castilla-La Mancha, 13001 Ciudad Real, Spain; 3 IMIBIC, Proteomic Unit, Maimonides Biomedical Research Institute of Cordoba, Reina Sofia University Hospital, University of Cordoba, 14071 Córdoba, Spain; 4 Neuroplasticity and Neurodegeneration Laboratory, Regional Centre for Biomedical Research, Ciudad Real Medical School, University of Castilla-La Mancha, 13001 Ciudad Real, Spain; 5 Faculty of Medicine, Institute of Neurosciences, Universidad Autónoma de Barcelona (UAB), 08193 Barcelona, Spain

**Keywords:** 3xTg-AD mice, Alzheimer’s disease, coenzyme Q10, MALDI-imaging, mass spectrometry, ubiquinol

## Abstract

Despite its robust proteopathic nature, the spatiotemporal signature of disrupted protein modules in sporadic Alzheimer’s disease (AD) brains remains poorly understood. This considered oxidative stress contributes to AD progression and early intervention with coenzyme Q10 or its reduced form, ubiquinol, delays the progression of the disease. Using MALDI–MSI and functional bioinformatic analysis, we have developed a protocol to express how deregulated protein modules arise from hippocampus and cortex in the AD mice model 3xTG-AD in an age-dependent manner. This strategy allowed us to identify which modules can be efficiently restored to a non-pathological condition by early intervention with ubiquinol. Indeed, an early deregulation of proteostasis-related protein modules, oxidative stress and metabolism has been observed in the hippocampus of 6-month mice (early AD) and the mirrored in cortical regions of 12-month mice (middle/late AD). This observation has been validated by IHC using mouse and human brain sections, suggesting that these protein modules are also affected in humans. The emergence of disrupted protein modules with AD signature can be prevented by early dietary intervention with ubiquinol in the 3xTG-AD mice model.

## 1. Introduction

Alzheimer’s disease (AD) is the most common form of dementia, accounting for up to 75% of all cases [1]. However, despite researchers’ efforts to understand the basis of sporadic AD, the spatio-temporal deregulation of biological processes in AD brains (other than Aβ deposition and formation of Tau tangles), remains unclear. From a functional point of view, the overlapping of these events hinders the understanding of the highly heterogeneous proteopathic nature of AD [2]. Closely linked to the proteopathy of the disease, oxidative stress emerges as a major modulator of proteostasis, especially at prodromic AD stages [3]. For instance, overexpression of Aβ induces oxidative stress, which promotes accumulation of Aβ in a feedback-looping cascade, leading to cognitive impairment and dementia through synaptic damage and further neuronal injuries [4].

This considered, anti-oxidative stress therapy is a promising means of slowing down the deleterious loop between oxidative stress and proteostasis [5]. Ubiquinol, the reduced form of coenzyme Q10 (CoQ10), is a well-known antioxidant and a bioenergetic modulator that showed significant beneficial effects against in vitro Aβ internalization [6], such as decreasing brain Aβ levels and attenuating neuroinflammation [7]. Indeed, several studies have pinpointed the protective effects of CoQ10/ubiquinol at inflammation [7] and hypoxia [8], which led to the quantification of CoQ10 levels in patients with dementia [3]. However, the full effects of CoQ10/ubiquinol on AD have not been fully elucidated.

Proteomics-based strategies constitute the best approach for studying the myriad of disrupted processes in different pathologies [9,10,11]. Among the spatial proteomic technique’s variants, MALDI mass spectrometry imaging (MALDI–MSI) is gaining interest among neurodegenerative disease researchers, due to its ability to obtain spatial information data while maintaining the cytoarchitecture of the original sample [12,13]. Despite its versatility, MALDI–MSI has been scarcely employed in AD, even though distribution of analytes is essential to comprehend the extent and localization of CNS-damaging processes [14,15] and to test the efficacy of drug treatments [16].

To investigate the spatio-temporal signatures of disrupted-functional processes in AD and its modulation by ubiquinol, we studied the peptidic profile of the 3xTg-AD mice model fed a standard or ubiquinol-supplemented diet, compared to Wild Type (WT)/non-Tg mice. Using further functional analysis tools, we identified the main biological processes, protein modules, regulated to ubiquinol in the 3xTg-AD mice brain at hippocampus and cortex after 4 or 10 m of supplementation.

Our results indicate a signature composed of proteostasis- and oxidative stress-related protein modules that were deregulated early (6-month mice) in the hippocampus and emerged later (12-month mice) in cortical regions. Ubiquinol prevented the deregulation and further advancement of protein modules signatures linked to AD progression in 3xTg-AD mice. Results were validated by immune-histochemistry in both mouse and human hippocampal and cortical brain sections.

## 2. Materials and Methods

### 2.1. Animals

Female triple transgenic AD (3xTgAD) mice harboring PS1/M146V, APPSwe and TauP301L transgenes (obtained from the colony established in the Medical Psychology Unit, School of Medicine, Autonomous University of Barcelona [17]), and age-matched nontransgenic (Non-Tg) controls with the same genetic background (C57BL/6x129/Sv) were group housed (2–4 per cage); these elements were maintained at 22 ± 2 °C and 50–70% relative humidity under a 12 h light and dark cycle. A selection of 2-month-old 3xTg-AD mice were randomly distributed according to the supplemented diet used. A standard diet (Teklad Global; Envigo, Huntingdon, UK) was fed to Vh Group, whereas a standard diet supplemented with 60 mg/100 g ubiquinol (prepared by Envigo from Teklad Global), named Ubi group. Non-Tg mice (wild type) were fed a standard diet (WT group). Animals (9 per group) received their prescribed diet and water ad libitum from 2 months until euthanasia (6 or 12 months).

Animal procedures followed European (Directive 2010/63/EU) and Spanish (RO 53/2013) legislation on the protection of animals used for scientific purposes. Experiments were previously approved by the Ethical Committee of Animal Research of the University of Castilla–La Mancha (PR-2017-02-02).

### 2.2. Behaviourial Tests

The open field and neophobia tests were performed at 6 or 12 months of age to study different behaviors and cognitive functions.

For the open field test, each mouse was released in one corner of a square plexiglass arena and allowed to freely explore the environment for 15 min. Movements of the mice were tracked, and the number of rearings per mouse was quantified as a measure of vertical activity. For the neophobia test, mice were introduced into the center of a new standard home cage (Makrolon, 35 × 45 × 25 cm) and the number of visited corners was recorded for a period of 30 s.

### 2.3. Processing of Samples

#### 2.3.1. Murine Samples

Mice were anesthetized with ketamine hydrochloride (Ketolar, 1.5 mL/kg, 75 mg/kg; Parke-Oavis, Madrid, Spain) and xylazine (Xilagesic, 0.5 mL/kg, 10 mg/kg; Calier, Barcelona, Spain), and perfused with a saline solution followed by 4% *w*/*v* paraformaldehyde in 0.1 M sodium phosphate buffer (pH 7.2). Whole brains were post-fixed in 4% *w*/*v* paraformaldehyde for 24 h, dehydrated and then embedded in paraffin blocks. Then, 5 μm sections were deparaffinized in xylene and rehydrated in a graded series of ethanol and de-ionized water.

#### 2.3.2. Human Samples

Postmortem human brain samples (N = 18) from neuropathologically diagnosed Alzheimer’s disease (AD) (N = 12) and non-Alzheimer’s disease (NAD) (N = 6) patients were used in the present study (Supplementary Table S1). Three experimental groups were established: N = 6 NAD cases, N = 6 AD (neuropathologically diagnosed as Braak tau stages III-IV) and N = 6 AD (neuropathologically diagnosed as Braak tau stages V–VI). Cases were provided by the August Pi I Sunyer Biomedical Research Institute (IDIBAPS: B.0000575), Murcia Region Network (BIOBANC–MUR: B.0000859), CIEN Foundation Tissue biobank (BTCIEN: B.0000741) and the Principality of Asturias biobank (BPA: B. 0000827), integrated into the Spanish National Biobanks Network; the cases were treated following standard operating procedures with the appropriate approval of the Ethical and Scientific Committees. All procedures described in this research were accredited by the Ethical Committee of Clinical Research of the Ciudad Real University Hospital (PID2019-108659RB-I00). Human samples for histological procedures were processed as previously reported [18]. For cryoprotection, tissue was embedded in a phosphate-buffered solution of 2% dimethyl sulfoxide (DMSO) and 10% glycerol for 48 h; it was subsequently embedded in a phosphate-buffered solution of 2% DMSO and 20% glycerol for 48 h. The tissues were covered in 30% sucrose and cut into 50 µm coronal sections with a freezing microtome.

### 2.4. MALDI Mass Spectrometry Imaging (MALDI–MSI)

#### 2.4.1. Sample Preparation

Paraffined tissues were cut at 5 µm of thickness and mounted onto an indium tin oxide slide (ITO, Sigma Aldrich, Steiheim, Germany) previously coated with poly-L-lysine (P1274-25 mg, Sigma Aldrich, Steiheim, Germany) with a custom protocol (0.1 mg/mL poly L-Lys, 37 °C, 1 h). Mounted samples were heated for 1 h at 65 °C to increase tissue fixation on ITO slide. Sections were then deparaffined in xylene and rehydrated in a graded series of ethanol and de-ionized water. Antigen retrieval steps were performed by heating the rehydrated samples in 10 mM sodium citrate pH 6 (AD7-950-270-0500, Enzo, Farmingdale, NY, USA) at 98 °C for 30 min. After cooling the samples to 40 °C at room temperature, we performed two washing steps with NH_4_HCO_3_ 10 mM buffer and a drying step in vacuum for 30 min prior to tissue digestion.

#### 2.4.2. On-Tissue Digestion and Matrix Deposition

On-tissue digestion of samples was performed by spraying 4 layers of trypsin (V5111, Promega, Madison, WI, USA) 0.1 µg/mL in 25 mM NH_4_HCO_3_, 10% TFE, at a flow of 10 mL/min with a SunCollect (Sunchrom, Germany) sprayer. Once the on-tissue digestion was finished, slides were dried at vacuum for 30 min before matrix deposition. MALDI matrices were deposited onto tissue using a solution based on α-cyano-4-hydroxycinnamic acid and CHCA (70990, Sigma Aldrich, Steiheim, Germany) (7 mg/mL 60% ACN 0.2% TFA). The internal calibrant Glu-Fibrinopeptide (Sigma Aldrich, Steiheim, Germany) was added to the matrix solution at a final concentration of 625 fmol/mL. The final matrix solution was sprayed in 8 layers: first layer at 10 mL/min, second layer at 20 mL/min, third layer at 30 mL/min, and the fourth to eighth layers at 45 mL/min; all layers were at a *z*-axis equal to 27.05. Once completed, slides were vacuum-dried for 30 min before analysis was performed on the mass spectrometer.

#### 2.4.3. Data Acquisition

Data were acquired with an AB 5800 TOF/TOF (ABSciex, Darmstadt, Germany). A positive reflector mode was used for all samples performing double internal calibration of the spectra using a trypsin autolysis peak an 842.508 [M + H]+ and a Glu-Fibrinopetide peak at 1570.677 [M + H]+. The *m*/*z* range for all samples was defined from 650 to 1800 (80% of visualized peptides from these samples were in this range—data not shown). The following parameters were fixed as default for all samples: laser intensity at 3200, delay extraction at 450 ns and the number of shots per pixel over the sample at 150. Twenty first shots were discarded to avoid sample background noise. Deflectors’ parameters were adjusted for each sample to ensure a resolution (FWHW) > 15,000 at GluFib mass. MSI datasets were acquired using TOF/TOF Imaging Acquisition Software (ABSciex) in a fixed spatial resolution of 150 μm for all samples. All mass spectrometry data have been deposited to the ProteomeXchange Consortium via the PRIDE repository [19] with the dataset identifier PXD037763.

#### 2.4.4. Pre-Processing Analysis of MALDI–MSI Data

Spectra data were loaded into the R statistical software (R Foundation for Statistical Computing). Cardinal MSI package was used for data processing. Data dimensionality reduction was performed with a resampling method that consisted of a linear interpolation at *m*/*z* unit using the two closest *m*/*z* points at which intensities were measured. Basic tools from the R programming language were used to obtain an average resampled spectrum of each ROI for subsequent classification and to perform, when indicated, a correlation analysis. Signal intensities were extracted and uploaded to MetaboAnalyst 4.0 [20] for statistical analysis. Datasets were previously filtered by Interquartile range (IQR) and, to make the assumptions fit correctly, data logarithmic transformation and auto-scaling were performed. Univariate analyses, such as fold-change analysis and t-test, were performed to compute summary-level statistics for each feature and individual study. Partial Least Squares for Discriminant Analysis (PLS-DA) was employed as a supervised learning method to represent the separation between both groups.

### 2.5. Protein Identification

Proteins were annotated according to the previously published and available database MaTisse [21]. Resolution of the database was adjusted to suit the features of the study; the matching protein mass was conducted individually and checked by two independent researchers. Matches with a difference of ±0.02 were excluded. All the proteins identified are described in Supplementary Tables S2–S9.

### 2.6. Functional Analysis and Protein–Protein Interaction Networks

For the functional enrichment study, proteins expressing statistically significant differences (*p* < 0.05) between WT and/or Ubi compared to Vh were identified and examined for relevant functional annotations using the Metascape tool (https://metascape.org/gp/index.html (accessed on 11 February 2023)). The functional annotations used were: GO terms (GO biological process, GO molecular function, GO cellular component), Reactome, and KEGG pathways. Protein–protein interaction analysis was performed using the STRINGApp plugin [22] with Cytoscape 3.9.1 software (https://cytoscape.org/ (accessed on 11 February 2023)). Interaction networks were identified using the default parameters, a confidence score of 0.4, and a maximum of 5 additional interactors. The clustering plugin clusterMaker [23] was also used to generate more readable and clustered networks. The granularity parameter used was 2.5. Additionally, the String Enrichment function of the STRINGApp plugin was used to identify meaningful functional annotations of those interaction networks with a minimum of 4 members. The functional annotations used were: GO terms (GO biological process, GO molecular function, GO cellular component), Reactome, and KEGG pathways.

### 2.7. Validation by Immunofluorescence

#### 2.7.1. Selection Criteria for Validation

The selection of markers for validation was based on the significance of their relative abundance in WT and/or Ubi compared to Vh, and their potential correlation to the etiology of AD. To assess the percent homology between the peptide sequences of the detected masses and their potential human equivalents, a sequence alignment with the Blast tool (https://blast.ncbi.nlm.nih.gov/Blast.cgi (accessed on 11 February 2023)) was performed, using the default parameters for each selected member. Those peptides whose alignments had a percentage of identity ≤90% were excluded for further validation.

#### 2.7.2. Immuno-Histochemistry and Imaging Protocol

For mice immunofluorescence, fixed, paraffin-embedded tissue was sectioned at 5 µm and deparaffined as explained above. Sections were washed in Phosphate-buffered saline (PBS) and blocked with 1% bovine serum albumin (BSA; Sigma-Aldrich A7284, St. Louis, MO, USA) and 0.5% Triton X-100 in PBS for 1 h at room temperature. The primary antibodies used are described in Supplementary Table S2. Sections were then washed and incubated with the appropriate secondary antibodies for 1 h at room temperature. To stain nuclei, brain sections were mounted in Vectashield medium with DAPI (Vector Laboratories, H-1200). A total of ten images of three different hippocampal or entorhinal cortex sections per animal were acquired on an LSM800 confocal microscope (Zeiss) using a 40× objective at room temperature. All analyses were performed in ImageJ using regions of interest on images to determine cell intensity in the respective fluorescence channels.

For human immunofluorescence, hippocampus and cortex sections were chosen. Tissue antigenicity was unmasked as described previously [24]. Subsequently, tissue was immersed in blocking buffer with 10% *v*/*v* normal donkey serum (Vector Laboratories, Burlingame, CA, USA), 0.1% Triton X-100 in Tris-buffered saline (TBS 0.05 M, pH 7.6) for 60 min at RT. Sections were incubated overnight at 4 °C with the following primary antibodies: Exportin-1/CRM1, ACAD9, eIF3a; (see Supplementary Table S2) in TBS with 0.3% Triton X-100. Sections were then incubated for 2 h at room temperature with Alexa-Fluor 488 (in TBS with 0.3% Triton X-100, see Supplementary Table S2), counterstained with DAPI (0.01% in TBS, Sigma Aldrich, St Louis, MO, USA) for 5 min, mounted, and coverslipped with PVA-DABCO (Sigma–Aldrich, St Louis, MO, USA). Three images of each section were acquired (*n* = 90) on an LSM800 confocal microscope (Zeiss) using a 63× oil objective at room temperature. All analyses were performed in ImageJ using regions of interest on images to determine cell intensity in the respective fluorescence channels. For the cresyl–violet Nissl stain, samples were incubated in 1% cresyl–violet solution for 15 min, cleared in 1% acetic acid for 1 min and rinsed in tap water for 5 min to remove excess stain. Slides were then washed in 70% ethanol for 1 min, dehydrated through 2 × 3 min changes of absolute ethanol, cleared in 2 changes of xylene for 5 min each, and coverslipped. Stereological analysis was performed using Stereo Investigator software (MBF Bioscience coupled with a Zeiss Axio Imager M2 microscope).

### 2.8. Statistical Analysis

Data are expressed as the mean ±SEM. Statistical analyses were carried out with GraphPad Prism v.8 software (GraphPad Inc., San Diego, CA, USA). The Mann–Whitney U test was performed on experimental data. Significant differences were considered at * *p* < 0.05; ** *p* < 0.01; *** *p* < 0.001.

## 3. Results

### 3.1. Behavioural Tests

Supplementary Figure S1 illustrates the variables assessed in the neophobia and openfield test, which were quantified by the number of visited corners and rearings, respectively. At 6 m of age, all the groups covered a similar number of visited corners (means: WT = 8.66; Vh = 8; Ubi = 8.33), though vertical exploration activity showed distinct patterns for Ubi mice and Vh mice (Vh vs. Ubi, *p* < 0.0476). Differences were found between WT and Vh mice groups (WT vs. Vh, *p* < 0.0303). At 12 m of age, significant differences were found in neither the open-field test (means: WT = 9.5; Vh = 14; Ubi = 9.8), nor in the neophobia test (means: WT = 5; Vh = 1.75; Ubi = 2.4).

### 3.2. MALDI–MSI Methodology

**Figure 1:**
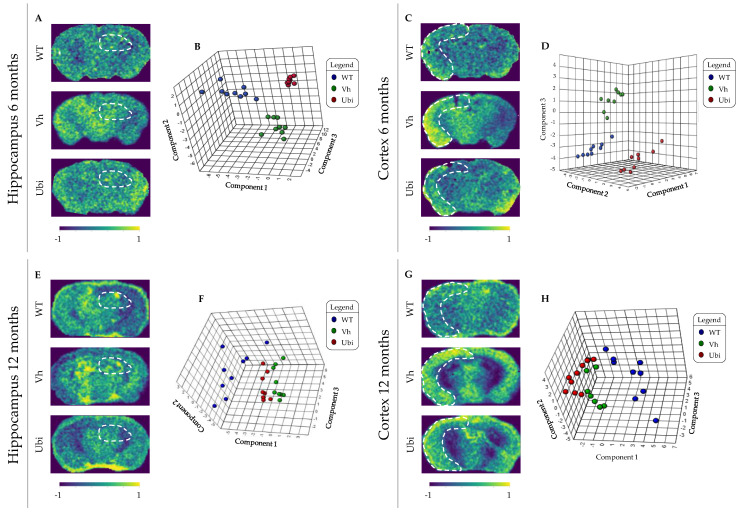
Peptide ion images and partial least-squares discriminant analysis (PLS-DA) score plots. (**A**,**C**,**E**,**G**) Representative molecular images of peptide ion detected at *m*/*z* 115,459; 96,657; 1391 and 1354, respectively. The ROIs in left and right brain sections correspond to hippocampal and cortical regions, respectively. PLS-DA 3D score plot analysis of hippocampus 6-m (**B**), cortex 6-m (**D**), hippocampus 12-m (**F**), and cortex 12-m (**H**). Results are shown as mean values of three replicates per mouse and three different mice per experimental groups, WT, Vh, and Ubi, respectively.

To determine the differences in spatial peptide distribution among experimental groups, specifically in hippocampal and cortical regions, MALDI–MSI was performed on paraffined coronal mice brain sections (Supplementary Figure S2). Analysis of brain sections uncovered a total of 57.501 individual *m*/*z* values; only 5000 features remained after the filtering and exclusion of poor-quality peaks according to interquartile range. Although no major differences were observed visually in signal intensity throughout the intensity map image (Figure 1A,C,E,G), the mass spectra of both regions showed a distinct peptide profile in 6 and 12 m mice (Supplementary Figures S3 and S4).

A supervised clustering analysis based on 3D PLS–DA was further performed to assess individuals in each experimental group. As shown in Figure 1B,D,F,H, three separated experimental groups were observed in 6 m hippocampus and cortex, displaying three well-clustered and independent groups. However, 3D PLS-DA for 12 m mice showed overlaps, particularly among Vh and Ubi groups. Therefore, heat-map representations of the top 100 significant features (Supplementary Figure S5) and top 25 correlation analysis (Supplementary Figure S6) confirmed the different profiles of tendencies in response to diet-induced changes.

### 3.3. Functional Analysis and Protein–Protein Interaction Analysis

**Figure 2:**
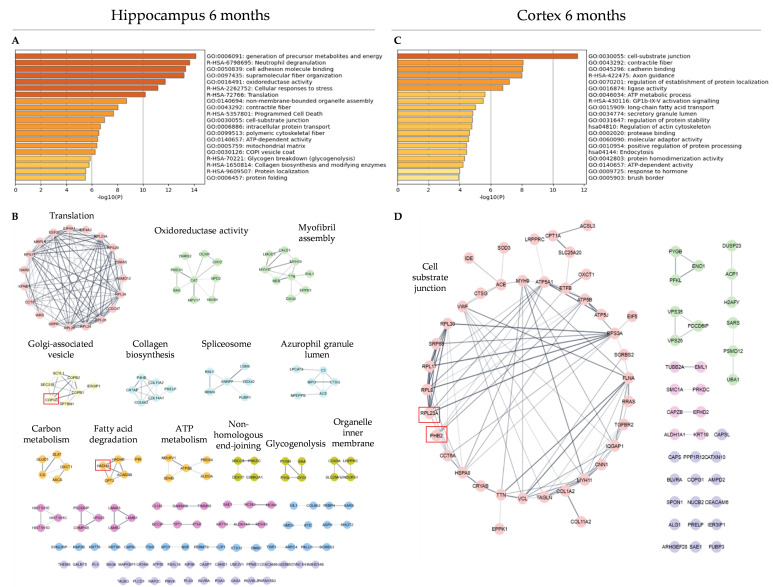
(**A**,**C**) Bar graph of the top 20 enriched terms across input gene lists, colored by *p*-values, in hippocampus 6 m and cortex 6 m, respectively. “Log10 (P)” is the *p*-value in log base 10. (**B**,**D**) Protein–protein interaction (PPI) network analysis of differentially expressed and selected genes using STRING database in hippocampus 6 m and cortex 6 m, respectively. Each module was analyzed by plug-in STRING Enrichment in Cytoscape, showing the most significative functional annotation on each case. Red-squared nodes refer to members selected for further validation.

**Figure 3:**
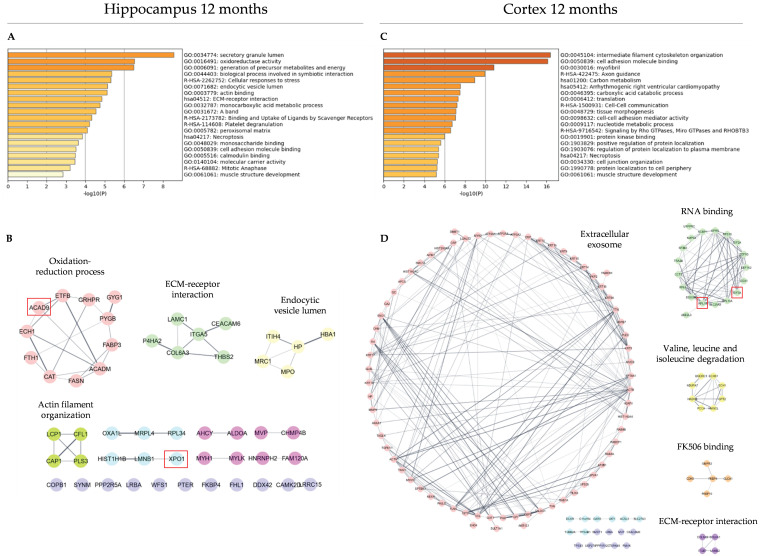
(**A**,**C**) Bar graph of the top 20 enriched terms across input gene lists, colored by *p*-values, in hippocampus 12 m and cortex 12 m, respectively. “Log10 (P)” is the *p*-value in log base 10. (**B**,**D**) Protein–protein interaction (PPI) network analysis of differentially expressed and selected genes using STRING database in hippocampus 12 m and cortex 12 m, respectively. Each module was analyzed by plug-in STRING Enrichment in Cytoscape, showing the most significant functional annotation on each case. Red-squared nodes refer to members selected for further validation.

Masses whose relative abundances were similar between the WT and Ubi groups, and different to the Vh group, were chosen for further functional analysis. Using a depurated database [21], the *m*/*z* value of each feature was translated into the name of the putative protein. In total, 478 proteins from the 188 masses studied that met the criteria, were included in the over-representation functional annotations analysis using the Metascape bioinformatics tool (Figure 2A,C and Figure 3A,C).

For 6 m hippocampus, the most significant top-three functional terms were supramolecular fiber organization (21 proteins); translation (15 proteins), and neutrophil degranulation (17 proteins) (Figure 2A); for 6 m cortex, the most significant top-three functional terms were structural molecule activity (12 proteins), actomyosin structure organization (6 proteins), and cadherin binding (7 proteins) (Figure 2C). In contrast, 12 m hippocampus was mainly enriched in proteins involved in actin filament-binding (6 proteins); RHO GTPase effectors (5 proteins), and focal adhesion (4 proteins) (Figure 3A); proteins related to structural molecule activity (25 proteins), cell adhesion molecule binding (19 proteins) and axon guidance (13 proteins) were enriched in 12 m cortex (Figure 3C).

Many proteins identified shared similar biological functions or signaling pathways, suggesting a functional relevance. For a thorough analysis, a STRING protein–protein interactions (PPIs) network was performed based on close- functional associations or physical interactions (Figure 2B,D and Figure 3B,D). In 6 m hippocampus, this strategy revealed a significant enrichment (*p* = 1.0 × 10^−16^) of PPIs (edges) among the 128 proteins included (Figure 2B). Most of the PPIs were focused on six main protein modules; RNA binding (e.g., RPL26, EEF2), redox processes (e.g., CAT, PRDX1), and assemble of myofibril (e.g., EPPK1, MYH11) were the most represented. However, one cluster related to cell–substrate junctions comprising 22 edges out of 40 proteins included could be found in 6 m cortex (*p* = 1.03 × 10^−10^) (Figure 2D). A lower but significative enrichment (*p* = 0.0328) was reported from 29 proteins included in 12-m hippocampus (Figure 3B), wherein two clusters involved in actin binding (e.g., FLNA, FHL1; 13 nodes) and structure of ribosomes (e.g., RPL13A, RPS11; 4 nodes) were highlighted. As in 6 m hippocampus, a significant enrichment in 12 m cortical region (*p* = 5.73 × 10^−10^) of PPIs was observed (Figure 3D). Out of 82 proteins included, seven clusters were identified and the most represented were those involved in cell morphogenesis (e.g., CA2, TXN; 20 nodes), protein translation (e.g., EIF3A, RPL15; 9 nodes), and fatty acid beta-oxidation (e.g., ACSL3, ECH1; 8 nodes).

### 3.4. Protein Modules Validation by Immunofluorescence

**Figure 4:**
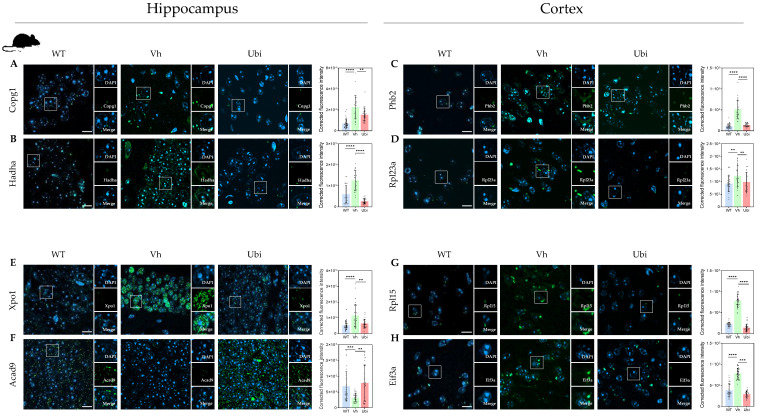
Protein expression of selected markers in mice sections. (**A**–**H**) Representative confocal images of selected markers in the hippocampus and cortical regions of 6 m and 12 m old brain mice sections. Scale bars: 200 μm. Graphs show mean values ± S.E.M. of corrected fluorescence intensities from 10 measurements from section, three sections per mice and three different mice for each experimental group, WT, Vh and Ubi, respectively. Significant differences were determined using the two-tailed Mann–Whitney test (** *p* < 0.01, *** *p* < 0.001, **** *p* < 0.0001).

We also selected a set of markers from mice brain sections (see Material and Methods) (Supplementary Table S11). For each region of study, two representative proteins are shown. At 6 m hippocampus, the expression of COPG1 and HADHA proteins was examined, given their important role in protein transport and fatty acid oxidation, respectively, (Figure 4A,B). Both COPG1 and HADHA significantly increased in Vh dentate gyrus compared to Ubi. In 6 m cortical region, the expression of PHB2 and RPL23A proteins, involved in mitophagy and ribosome structure, was analyzed (Figure 4C,D). A significant reduction in cortex for both PHB2 and RPL23A markers was also evident in Ubi group compared to Vh.

The expression of XPO1 in hippocampal regions of 12 m mice increased significantly in dentate gyrus in the Vh group compared to WT, whereas Ubi supplementation restored XPO1 expression to WT level (Figure 4E). While no differences in ACAD9 expression were found in dentate gyrus among the three groups, CA2/CA3 regions exhibited higher expression in WT and Ubi compared to Vh (Figure 4F). In cortical regions of 12 m mice, RPL15 and EIF3A expression was significantly downregulated by Ubi supplementation compared to Vh (Figure 4G,H).

**Figure 5:**
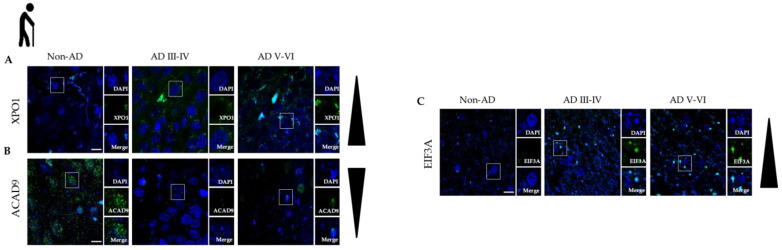
Protein expression of selected markers in human sections. (**A**–**C**) Representative confocal images of selected markers in the hippocampus (left) and cortical regions (right) of XPO1 (**A**), ACAD9 (**B**) and EIF3A (**C**) in human brain samples. Scale bars: 200 μm.

Three candidate proteins, ACAD9, EIF3A and XPO1, were assessed in human AD and healthy donors’ samples by immunofluorescence, using brain regions homologous to those studied in mice (Figure 5A–C). In addition, early (Braak stage III-IV) and late (Braak stage V-VI) AD samples were analyzed to evaluate the diagnostic validity of our markers at different stages of the disease.

As shown in Figure 1, staining intensity was detected in AD hippocampus from both stages compared to non-AD hippocampus (Figure 5A). Inversely, ACAD9 protein levels were higher in non-AD hippocampus compared to a low-to-zero expression in both early- and late-stage AD samples (Figure 5B). Finally, EIF3A exhibited a granular expression pattern in early- and late-stage AD cortex, but not in non-AD cortex wherein the immunostaining was hardly visible (Figure 5C).

## 4. Discussion

Alzheimer’s disease (AD) is the leading cause of dementia and is characterized by a gradual decline in cognitive function, which typically begins with widespread oxidative damages in several regions of the brain. This considered, the lipophilic antioxidant CoQ10 has been so far investigated for its potential neuroprotective ability at delaying AD progression [6,7]. Even though oxidative stress is intimately linked to proteostasis, how CoQ10 regulates the proteostasic balance has not been studied to date.

In this study, we describe the main protein modules deregulated by AD in 3xTgAD mice and the spatio-temporal progression of these deregulated modules to establish protein signatures that mark progression from initial to advanced stages of the disease. The protein modules obtained by MALDI–MSI have been validated by immuno-histochemical techniques in both mice and human brain sections.

A diet supplemented with Ubi generated substantial alterations in the peptide profile of the hippocampus and cortex of 3xTgAD mice (Supplementary Figures S3–S6). PLS–DA analysis established a significant difference between WT, Vh and Ubi peptide profiles at 6 m (Figure 1B,D). Nevertheless, Vh and Ubi clusters showed several overlaps at 12 m (Figure 1F,H). This finding might suggest that Ubi is most effective on early AD, probably because it primarily targets oxidative damages associated with premorbid stages of the disease.

To study the biological processes deregulated with the disease and prevented by Ubi-supplemented diet, a functional analysis was performed with Metascape (Figure 2A,C and Figure 3A,C). Hippocampus constitutes one of the first brain regions affected by AD [3]. In this sense, a high number of biological deregulated processes were found in 6 m mice, resembling the affectation of this structure during AD progression (Figure 2A,B). These major protein modules altered in hippocampus are related to structural changes (i.e., myofibril assembly, collagen biosynthesis), metabolism (i.e., carbon metabolism and ATP metabolism), and protein synthesis machinery (i.e., protein translation, Golgy-associated vesicles, spliceosome), as shown in previously published data [25]. Though at this stage the disease has not greatly damaged other brain regions, a main protein module related to cell substrate junctions was altered in the cortex of 6 m mice, as observed in early preclinical AD [26] (Figure 2C,D).

An opposite landscape has been observed at 12 months, when the disease is clearly stablished. At this advanced stage, a high affectation was inferred from cortical regions, denoted by a high number of protein modules affected compared to the hippocampus. However, in hippocampal regions of 12 m mice (Figure 3A,B) protein modules related to redox reactions and ECM–receptor interaction and actin filament organization were found. These findings are indicative of extensive and irreparable hippocampal damage at this advanced AD stage [3]. On the other hand, in the cortical region of 12 m mice (Figure 3C,D) a wider number of protein modules, such as RNA binding or BCAAs degradation, were altered; this resembled the functional scenario previously observed in hippocampal regions at 6 months. This indicates a gradual increase in deregulated protein modules related to proteostasis and metabolism from hippocampus to cortex during AD progression.

Previous studies not only found similar functional modules (such as ATP metabolism or glycolysis) [1,2], but also particular indicators that are reflected in our study (i.e., NDUF, ATP5B or ALDOA) [3,4]. For example, proteomics of mitochondria and endoplasmic reticulum fractions in 3xTg-AD astrocytes, has revealed changes in cell adhesion and protein production that are consistent with our findings [5]. Additionally, other antioxidants such as vitamin A [6] produced effects resembling those found with ubiquinol, thereby raising the relevance of antioxidant therapy as a modulator of the functional impairment present in AD.

The two proteins of each region and the state of the altered identified protein modules were further validated to verify the protective role of Ubi in AD. Since AD is characterized by dysregulated proteostasis and mitochondrial dysfunction at early stages, two proteins (COPG1 and HADHA–components of proteostasis machinery and mitochondrial beta-oxidation pathway, respectively), were selected for 6 m hippocampus. Both correlated with the progression of AD and were upregulated in Vh group vs. WT and ubiquinol restored to WT levels (Figure 4A,B). While no evidence regarding the relationship between COPG1 and the development of AD has been shown, Wang et al. showed that HADHA protein presented a similar expression pattern to Aβ42, with increased expression in cortex and decreased expression in cerebrospinal fluid from AD patients [7]. PHB2, a membrane-bound chaperone involved in the correct folding and assembly of components of the mitochondrial respiratory chain [27], and RPL23A, a component of the large ribosomal subunit, exhibited similar expressions in Ubi and WT groups (Figure 4C,D). These findings show that ubiquinol partially reverts the proteostatic and redox systems to functioning towards a more WT-like profile.

XPO1 and ACAD9 were selected to validate the results obtained in 12 m hippocampus (Figure 4E,F). XPO1 participates in nuclear protein transport and enhanced levels have been related to increased risk of late-onset AD [28]. Elevated levels of XPO1 were found in the hippocampal region in both human (Figure 5A) and mice (Figure 4E) sections, whose expression was downregulated to WT levels in mice after the Ubi diet.

ACAD9 catalyzes α-β dehydrogenation of fatty acyl-CoA thioesters [29] and showed downregulated expression in Vh compared to WT; this was restored by ubiquinol in mice (Figure 4F). We also observed an AD progression-related decrease in ACAD9 levels in human brains (Figure 5B). Though no evidence currently exists to suggest a direct link between ACAD9 and AD, this protein is involved in the formation of the mitochondrial complex I and related to bioenergetics; its decrease can trigger an increase in oxidative stress [29], which is associated with AD progression.

In 12 m cortex, the upregulated expression of RPL15, a component of the large ribosomal subunit [30], and EIF3A, a component of the eIF-3 complex required for the initiation of protein synthesis [31], in the Vh group was restored to WT level in Ubi group (Figure 4G,H). As in mice, our results show that EIF3A was increased in human AD brains; its expression might be linked to the progression of the disease (Figure 5C). This evidence supports the existence of a late engagement of the cortex’s proteostatic and metabolic machinery, as previously shown in the hippocampus at early stages. Since ubiquinol partially reversed these dysregulated protein modules, we propose longer-term investigations to explore its pathological efficacy.

In summary, our study revealed a relationship between both early hippocampal and later cortical affectation of protein modules, and oxidative stress, proteostasis, and bioenergetics; this relationship can be prevented by intervention with ubiquinol in diet.

## 5. Conclusions

Our methodology introduces a systematic strategy to identify protein modules signatures of AD progression, which could serve as a diagnostic AD-staging tool. With this strategy we also demonstrated that ubiquinol acts as an effective preventive molecule when administered early, before the onset of the disease, by impeding or dampening the deregulation of proteostasis, redox machinery and metabolism.

## Data Availability

The source data for each figure are available from the corresponding author on reasonable request. All mass spectrometry data have been deposited in the ProteomeXchange Consortium via the PRIDE repository29 with the dataset identifier PXD037763.

## Supplementary Materials

The following supporting information can be downloaded at: https://www.mdpi.com/article/10.3390/antiox12030747/s1: Supplementary material file, containing Supplementary Tables S1–S11, Supplementary Figures S1–S6.
