# Supplementary material for: Spatial and Temporal Protein Modules Signatures Associated with Alzheimer Disease in 3xTg-AD Mice Are Restored by Early Ubiquinol Supplementation
Source: Antioxidants (Basel). 2023 Mar 19;12(3):747. doi: 10.3390/antiox12030747 (PMC10044705; doi:10.3390/antiox12030747)
Supplement: Supplementary file 1 [file antioxidants-12-00747-s001.zip › antioxidants-2246880-supplementary.pdf]

Table S1. Demographic and clinicopathological features of the patients used in the study.

Patient number	Study area	Diagnosis	Sex	Age	PMD (hh:mm)	Brain weight (grs)	Braak tau stage	Disease duration (years)	Cause of death
1	Hippocampus	NP	F	62	2:00	1050	-	-	Cardiorespiratory arrest (immediate), multi-organic failure (secondary)
2		NP	F	59	2:00	1200	-	-	Severe acute pancreatitis (immediate), cardiorespiratory arrests (secondary)
3		NP	F	58	5:00	944	0	-	Pneumonia
4		AD	F	78	5:50	1137	III	m.d.	m.d.
5		AD	F	84	17:00	1260	III-IV	4	Cardiogenic shock; electrolyte disturbance
6		AD	F	69	8:20	m.d.	IV	m.d.	Respiratory insufficiency
7		AD	F	71	10:00	1006	V	12	m.d.
8		AD	F	79	3:00	950	VI	11	m.d.
9		AD	F	63	5:00	1000	VI	9	Cardiorespiratory arrest
10	Cortex	NP	F	62	2:00	1050	-	-	Cardiorespiratory arrest (immediate), multi-organic failure (secondary)
11		NP	F	59	2:00	1200	-	-	Severe acute pancreatitis (immediate), cardiorespiratory arrests (secondary)
12		NP	F	58	5:00	944	0	-	Pneumonia
13		AD	F	78	5:50	1137	III	m.d.	m.d.
14		AD	F	90	12:20	1175	III	m.d.	Cerebral haemorrhage
15		AD	F	84	17:00	1260	III-IV	4	Cardiogenic shock; electrolyte disturbance
16		AD	F	87	5:00	1000	V	3	Cardiorespiratory arrest (immediate), Alzheimer's disease (secondary)
17		AD	F	85	2:00	1150	V	5	Cardiorespiratory arrest (immediate), senile dementia (secondary)
18		AD	F	78	0,083333333	1000	V	3	Respiratory failure (immediate), community pneumonia (secondary)

AD: Alzheimer disease: NP: non pathology; F: female; m.d.: missing data.

Table S2. Protein inference after *m/z* filtering from heatmap hippocampus 6m

Mass (*m/z*)	Near Mass Match	Gene name	Protein name
702,40	702,41	LRPPRC	Leucine-rich PPR motif-containing protein, mitochondrial
1050,55	1050,54	PMVK	Phosphomevalonate kinase
	1050,54	C8ORF55	UPF0670 protein C8orf55
	1050,55	NDUFV1	Isoform 1 of NADH dehydrogenase [ubiquinone] flavoprotein 1, mitochondrial
	1050,56	CSNK2A1	Casein kinase II subunit alpha'
	1050,56	ABHD14B	Isoform 1 of Abhydrolase domain-containing protein 14B
	1050,56	TTN	Titin isoform N2-A
	1050,56	SPTBN1	Isoform Long of Spectrin beta chain, brain 1
	1050,56	LMOD1	Isoform 1 of Leiomodin-1
1063,55	1063,52	PSMA5	Proteasome subunit alpha type-5
	1063,52	d.n.f	cDNA FLJ61158, highly similar to ADP-ribosylation factor-like protein 8B
	1063,55	d.n.f	Similar to complement component C3, partial
	1063,55	d.n.f	Complement C3 (Fragment)
	1063,56	CPT2	Carnitine O-palmitoyltransferase 2, mitochondrial
	1063,56	CRTAP	Cartilage-associated protein
	1063,56	HAGH	Isoform 1 of Hydroxyacylglutathione hydrolase, mitochondrial
1153,60	1153,59	d.n.f	cDNA FLJ56452, highly similar to Echinoderm microtubule-associated protein-like 2
	1153,59	OAS3	2'-5'-oligoadenylate synthetase 3
	1153,60	RPL34	60S ribosomal protein L34
	1153,60	COPB2	Coatomer subunit beta'
	1153,60	MPV17	Isoform 1 of Mitochondrial inner membrane protein
	1153,60	MPV17	Isoform 1 of Mitochondrial inner membrane protein
	1153,60	FTH1	Ferritin heavy chain
	1153,60	CHMP4B	Charged multivesicular body protein 4b
	1153,61	RALY	RNA binding protein, autoantigenic (HnRNP-associated with lethal yellow homolog (Mouse)), isoform CRA_a (Fragment)
	1153,61	MCM4	DNA replication licensing factor MCM4
1054,55	1054,54	SNRPF	Small nuclear ribonucleoprotein F
	1054,55	CASP7	Isoform Alpha of Caspase-7
	1054,55	SCYL1	Isoform 4 of N-terminal kinase-like protein
	1054,55	d.n.f	cDNA FLJ41755 fis, clone HSYRA2009102, highly similar to Adenosine 3'-phospho 5'-phosphosulfate transporter 1
	1054,55	PGA3	Pepsinogen 3, group I
	1054,56	d.n.f	cDNA FLJ46199 fis, clone TESTI4007965, highly similar to AP-1 complex subunit gamma-1
	1054,56	d.n.f	cDNA FLJ46199 fis, clone TESTI4007965, highly similar to AP-1 complex subunit gamma-1
	1054,56	RPS11	40S ribosomal protein S14
	1054,56	CRYZ	Quinone oxidoreductase
1040,55	1040,54	HADHA	Trifunctional enzyme subunit alpha, mitochondrial
	1040,55	DHRS2	Dehydrogenase/reductase member 2 isoform 2
	1040,55	ITIH5	Inter-alpha trypsin inhibitor heavy chain precursor 5 isoform 1
	1040,55	NPEPPS	Puromycin-sensitive aminopeptidase
1039,55	1039,53	RPL26	60S ribosomal protein L26
	1039,53	EEF2	Elongation factor 2
	1039,55	d.n.f	cDNA FLJ55809
	1039,56	DYNC1H1	Cytoplasmic dynein 1 heavy chain 1
1068,55	1068,55	EIF4A2	Isoform 1 of Eukaryotic initiation factor 4A-II
	1068,55	EIF4A1	Eukaryotic initiation factor 4A-I
1022,55	1022,54	STX12	Syntaxin-12
	1022,55	PALLD	Isoform 4 of Palladin
	1022,56	d.n.f	cDNA, FLJ96508, Homo sapiens SH3-domain GRB2-like 1 (SH3GL1), mRNA
1021,55	1021,54	COL6A2	Isoform 2C2 of Collagen alpha-2(VI) chain
	1021,55	FLII	Protein flightless-1 homolog
	1021,55	MCM2	DNA replication licensing factor MCM2
	1021,55	GLUD1	Glutamate dehydrogenase 1, mitochondrial
	1021,56	DDX17	Isoform 4 of Probable ATP-dependent RNA helicase DDX17
851,60	N/A	d.n.f	d.n.f
850,60	N/A	d.n.f	d.n.f
1113,65	1113,64	MYH11	Myosin-11
	1113,65	CCDC47	Isoform 1 of Coiled-coil domain-containing protein 47
	1113,65	LAMB2	Laminin subunit beta-2
	1113,66	BAG6	Isoform 1 of Large proline-rich protein BAT3
	1113,66	d.n.f	482 kDa protein
	1113,66	GYG1	Isoform GN-1L of Glycogenin-1

1295,70	1295,68	CAND1	Isoform 1 of Cullin-associated NEDD8-dissociated protein 1
	1295,68	KPNB1	Importin subunit beta-1
	1295,69	MAPKBP1	Isoform 1 of Mitogen-activated protein-binding protein-interacting protein
727,45	727,45	VIL1	Villin-1
	727,45	LAMA5	Laminin subunit alpha-5
1175,70	1175,68	ARPC4	Actin-related protein 2/3 complex subunit 4
	1175,69	TTN	Titin isoform N2-A
971,60	971,59	COL14A1	Isoform 1 of Collagen alpha-1(XIV) chain
	971,59	GALNT5	Polypeptide N-acetylgalactosaminyltransferase 5
	971,59	RAP2B	Ras-related protein Rap-2b
	971,59	RAP2C	Ras-related protein Rap-2c
	971,59	ALDOA	Fructose-bisphosphate aldolase A
	971,59	CLUH	KIAA0664 Protein
	971,60	FBXL18	F-box/LRR-repeat protein 8
	971,61	EEF2	Elongation factor 2
	971,61	SRPRA	Signal recognition particle receptor subunit alpha
764,40	764,40	GMDS	GDP-mannose 4,6 dehydratase
	764,41	IARS1	Isoleucyl-tRNA synthetase, cytoplasmic
1155,60	1155,59	TTN	Titin isoform N2-A
	1155,60	RUVBL2	RuvB-like 2
	1155,61	PLCD1	1-phosphatidylinositol-4,5-bisphosphate phosphodiesterase delta-1
865,50	865,51	DNM2	Isoform 1 of Dynamin-2
1435,75	1435,74	KRT6B	Keratin, type II cytoskeletal 6B
	1435,74	HNRNPG	Heterogeneous nuclear ribonucleoprotein G
	1435,74	DARS1	Aspartyl-tRNA synthetase, cytoplasmic
	1435,75	ATP5F1B	ATP synthase subunit beta, mitochondrial
	1435,75	GSDMB	Isoform 3 of Gasdermin-B
	1435,75	PAFAH1B3	Platelet-activating factor acetylhydrolase IB subunit gamma
	1435,76	d.n.f	HLA class II histocompatibility antigen, DR alpha chain
	1435,76	d.n.f	UPF0727 protein C6orf115
	1435,76	TPT1	Tumor protein, translationally-controlled 1
1110,60	1110,59	d.n.f	265 kDa protein
	1110,59	SAMM50	Sorting and assembly machinery component 50 homolog
	1110,60	DCXR	L-xylulose reductase
	1110,60	SERPINA1 A	Isoform 1 of Alpha-1-antitrypsin
1161,60	1161,59	COL14A1	Isoform 1 of Collagen alpha-1(XIV) chain
	1161,59	NDUFS1	NADH-ubiquinone oxidoreductase 75 kDa subunit
	1161,59	d.n.f	Isoform 1 of Protein KIAA1967
	1161,59	AACS	Isoform 1 of Acetoacetyl-CoA synthetase
	1161,61	LPCAT3	Lysophospholipid acyltransferase 5
833,45	833,45	FHL1	Isoform 1 of Four and a half LIM domains protein 1
	833,46	DLAT	Dihydrolipoyllysine-residue acetyltransferase component of pyruvate dehydrogenase complex, mitochondrial
	833,46	GPD2	Isoform 1 of Glycerol-3-phosphate dehydrogenase, mitochondrial
	833,46	d.n.f	Putative uncharacterized protein PSME2
1339,75	1339,76	DDX42	Isoform 1 of ATP-dependent RNA helicase DDX42
	1339,76	PRKDC	Isoform 1 of DNA-dependent protein kinase catalytic subunit
920,50	920,49	d.n.f	cDNA FLJ53324, highly similar to Tight junction protein ZO-2
	920,51	PRDX1	Peroxiredoxin-1
	920,51	RPS29	40S ribosomal protein S29
	920,51	DSG2	Desmoglein-2
	920,51	PRDX4	Peroxiredoxin-4
1109,60	1109,59	COPG1	Coatomer subunit gamma
	1109,60	PYGM	Glycogen phosphorylase, muscle form
	1109,61	RHOT2	Isoform 1 of Mitochondrial Rho GTPase 2
	1109,61	XRCC5	ATP-dependent DNA helicase 2 subunit 2
1340,75	1340,74	MPO	Isoform H17 of Myeloperoxidase
	1340,74	MRPL4	Isoform 1 of 39S ribosomal protein L4, mitochondrial
1453,80	1453,79	CCT8	T-complex protein 1 subunit theta
	1453,79	TWF1	Isoform 3 of Twinfilin-1
	1453,80	NEB	Nebulin isoform 1
1154,60	1154,59	COL6A3	COL6A3 protein
	1154,59	COL6A3	Isoform 1 of Collagen alpha-3(VI) chain
	1154,60	CAT	Catalase
	1154,60	LCP1	Plastin-2
	1154,60	PLS3	Plastin-3
726,45	726,45	BAX	Isoform Alpha of Apoptosis regulator BAX
	726,45	CAND1	Isoform 1 of Cullin-associated NEDD8-dissociated protein 1

921,50	921,50	CRYAB	Alpha-crystallin B chain
	921,50	PSMD12	26S proteasome non-ATPase regulatory subunit 12
	921,50	BLVRA	Biliverdin reductase A
	921,50	OXCT1	Succinyl-CoA:3-ketoacid-coenzyme A transferase 1, mitochondrial
	921,52	RPL28	60S ribosomal protein L28
	921,52	MCM4	DNA replication licensing factor MCM4
	921,52	ACADSB	Short/branched chain specific acyl-CoA dehydrogenase, mitochondrial
	921,52	KIF5B	Kinesin-1 heavy chain
738,40	NO ID	d.n.f	d.n.f
701,45	701,46	UBE2V1	Isoform 3 of Ubiquitin-conjugating enzyme E2 variant 1
	701,46	FUBP1	Isoform 1 of Far upstream element-binding protein 1
990,55	990,54	IER3IP1	Immediate early response 3-interacting protein 1
	990,54	COL11A2	Isoform 1 of Collagen alpha-2(XI) chain
	990,56	CAPSL	Calcyphosin
	990,56	SARS	Seryl-tRNA synthetase
	990,57	HADHB	Trifunctional enzyme subunit beta, mitochondrial
	990,57	GAA	Lysosomal alpha-glucosidase
1046,60	N/A	d.n.f	d.n.f
966,55	966,54	RPL24	19 kDa protein
	966,54	RDH10	Retinol dehydrogenase 10
	966,57	P4HB	Protein disulfide-isomerase
	966,57	d.n.f	Putative uncharacterized protein P4HB
1064,60	1064,59	PRELP	Prolargin
	1064,59	VPS25	Vacuolar protein-sorting-associated protein 25
	1064,60	PDCD6IP	Programmed cell death 6-interacting protein
	1064,60	EPPK1	Epiplakin 1
	1064,60	RPL23A	60S ribosomal protein L23a
	1064,60	ALDH1A1	Retinal dehydrogenase 1
	1064,61	KRT19	Keratin, type I cytoskeletal 19
	1064,61	TTN	Titin isoform N2-A
	1064,61	SORBS2	Sorbin and SH3 domain containing 2 isoform 6

d.n.f: data not found

Table S3. Protein inference after *m/z* filtering from top25 hippocampus 6m

Mass (*m/z*)	Near Mass Match	Gene name	Protein name
726,80	726,45	BAX	Isoform Alpha of Apoptosis regulator BAX
	726,45	CAND1	Isoform 1 of Cullin-associated NEDD8-dissociated protein 1
973,60	973,60	H1-2	Histone H1.2
	973,60	H1-4	Histone H1.4
	973,60	H1-3	Histone H1.3
	973,60	NSF	Vesicle-fusing ATPase
1153,60	1153,60	RPL34	60S ribosomal protein L34
	1153,60	TIMM50	Isoform 1 of Mitochondrial inner membrane protein
	1153,60	COPB1	Coatomer subunit beta
	1153,60	FTH1	Ferritin heavy chain
	1153,60	CHMP4B	Charged multivesicular body protein 4b
808,4	808,41	ACE	Isoform Somatic-1 of Angiotensin-converting enzyme
	808,41	CTSG	Cathepsin G
950,50	950,51	SYNJ2BP	Synaptojanin-2-binding protein
938,50	938,51	MYH11	Myosin-11
	938,51	MYH10	Isoform 1 of Myosin-10
	938,51	XPO7	Exportin 7 isoform c
1063,55	1063,55	d.n.f	Similar to complement component C3, partial
	1063,55	C3	Complement C3 (Fragment)
834,45	834,45	SDH4	Succinate dehydrogenase [ubiquinone] cytochrome b small subunit, mitochondrial
1028,60	1028,60	FERMT3	Isoform 2 of Fermitin family homolog 3
1002,65	1002,63	PIR	Pirin
	1002,63	BCCIP	Isoform 1 of BRCA2 and CDKN1A-interacting protein
943,65	943,58	COX5A	Cytochrome c oxidase subunit 5A, mitochondrial
969,60	969,61	TAOK3	Serine/threonine-protein kinase TAO3
	969,61	PPME1	Isoform 2 of Protein phosphatase methylesterase 1
1615,8	1615,80	KRT78	Isoform 1 of Keratin, type II cytoskeletal 78
723,4	723,45	ATP5F1E	ATP synthase subunit alpha, mitochondrial
992,70	992,58	d.n.f	cDNA FLJ35730 fis, clone TESTI2003131, highly similar to ALPHA-1-ANTICHYMOTRYPSIN
900,60	900,59	CALD1	Isoform 1 of Caldesmon
887,55	887,53	ASPH	Aspartyl/asparaginyl beta-hydroxylase
813,40	813,41	FKBP4	FK506-binding protein 4
1359,85	1359,80	PRDX1	Peroxiredoxin-1
1108,70	1108,72	LSM6	U6 snRNA-associated Sm-like protein LSm6
1082,65	1082,67	LAMB1	Laminin subunit beta-2
834,50	834,51	CS	Citrate synthase, mitochondrial
1355,8	1355,79	PYGL	Glycogen phosphorylase, liver form
	1355,79	SLC25A12	Calcium-binding mitochondrial carrier protein Aralar1
	1355,79	ATIC	Bifunctional purine biosynthesis protein PURH
741,50	741,52	PSMD12	26S proteasome non-ATPase regulatory subunit 12
	741,52	SAE1	SUMO-activating enzyme subunit 1
977,55	977,54	SEC31A	Isoform 3 of Protein transport protein Sec31A

d.n.f: data not found

Table S4. Protein inference after *m/z* filtering from heatmap cortex 6m

Mass (*m/z*)	Near Mass Match	Gene name	Protein name
1202,65	1204,64	PHB2	Prohibitin-2
	1204,64	CNN1	Calponin-1
	1204,64	RPL17	60S ribosomal protein L17
	1204,64	d.n.f	149 kDa protein
	1204,64	NUCB2	Isoform 1 of Nucleobindin-2
	1204,66	MACROH2A1	Isoform 3 of Core histone macro-H2A.1
	1204,66	EIF5	Eukaryotic translation initiation factor 5
990,55	990,54	IER3IP1	Immediate early response 3-interacting protein 1
	990,54	COL11A2	Isoform 1 of Collagen alpha-2(XI) chain
	990,56	CAPSL	Calcyphosin
	990,56	SARS	Seryl-tRNA synthetase
838,45	838,45	VCL	Isoform 1 of Vinculin
	838,44	d.n.f	149 kDa protein
	838,47	AMPD2	Adenosine monophosphate deaminase 2
	838,47	SRP68	Isoform 1 of Signal recognition particle 68 kDa protein
724,50	N/A	d.n.f	d.n.f
1008,60	1008,59	d.n.f	cDNA FLJ44241 fis, clone THYMU3008436, highly similar to 6-phosphofructokinase, muscle type
	1008,59	PFKL	Isoform 1 of 6-phosphofructokinase, liver type
	1008,59	SERPINA1A	Isoform 1 of Alpha-1-antitrypsin
808,35	N/A	d.n.f	d.n.f
858,45	858,46	HSPA8	Isoform 1 of Heat shock cognate 71 kDa protein
	858,47	SLC25A20	Mitochondrial carnitine/acylcarnitine carrier protein
1064,60	1064,60	PDCD6IP	Programmed cell death 6-interacting protein
	1064,60	EPPK1	Epiplakin 1
	1064,60	RPL23A	60S ribosomal protein L23a
	1064,60	ALDH1A1	Retinal dehydrogenase 1
	1064,61	KRT19	Keratin, type I cytoskeletal 19
	1064,61	TTN	Titin isoform N2-A
	1064,61	SORBS2	Sorbin and SH3 domain containing 2 isoform 6
	1064,59	PRELP	Prolargin
	1064,59	VPS25	Vacuolar protein-sorting-associated protein 25
1262,65	1262,65	ATXN10	Ataxin-10
	1262,65	EFHD2	EF-hand domain-containing protein D2
	1262,65	TTN	Titin isoform N2-A
	1262,66	PRKDC	Isoform 1 of DNA-dependent protein kinase catalytic subunit
	1262,66	MYH9	Isoform 1 of Myosin-9
	1262,66	CCT6A	T-complex protein 1 subunit zeta
	1262,66	MYH11	Myosin-11
	1262,66	CCT6A	T-complex protein 1 subunit zeta
	1262,66	ACP1	Isoform 1 of Low molecular weight phosphotyrosine protein phosphatase
	1262,64	CAPZB	Isoform 2 of F-actin-capping protein subunit beta
	1262,64	ATP5F1B	ATP synthase subunit beta, mitochondrial
1355,65	1355,64	d.n.f	59 kDa protein
	1355,64	ACSL3	Long-chain-fatty-acid--CoA ligase 3
	1355,66	PPP1R12C	Isoform 1 of Protein phosphatase 1 regulatory subunit 12C
	1355,66	TUBB2A	Tubulin beta-2A chain
	1355,66	TTN	Titin isoform N2-A
808,4	808,41	ACE	Isoform Somatic-1 of Angiotensin-converting enzyme
	808,41	CTSG	Cathepsin G

d.n.f: data not found

Table S5. Protein inference after *m/z* filtering from top25 cortex 6m

Mass (*m/z*)	Near Mass Match	Gene name	Protein name
808,35	808,38	ENO1	Isoform alpha-enolase of Alpha-enolase
1355,65	1355,64	d.n.f	59 kDa protein
	1355,64	FAA3	Long-chain-fatty-acid--CoA ligase 3
	1355,66	PPP1R12C	Isoform 1 of Protein phosphatase 1 regulatory subunit 12C
	1355,66	TUBB2A	Tubulin beta-2A chain
	1355,66	TTN	Titin isoform N2-A
858,45	858,46	HSPA8	Isoform 1 of Heat shock cognate 71 kDa protein
808,40	808,41	ACE	Isoform Somatic-1 of Angiotensin-converting enzyme
	808,41	CTSG	Cathepsin G
1202,65	1202,65	ARHGEF28	Isoform 1 of Rho guanine nucleotide exchange factor 2
	1202,65	d.n.f	Putative uncharacterized protein KIF13B
1008,6	1008,67	VWF	Isoform 1 of von Willebrand factor A domain-containing protein 1
1354,65	1354,65	EML1	Isoform 1 of Echinoderm microtubule-associated protein-like 1
971,50	971,50	RPS3A	40S ribosomal protein S3a
1262,65	1262,65	ATXN10	Ataxin-10
	1262,65	EFHD2	EF-hand domain-containing protein D2
	1262,65	TTN	Titin isoform N2-A
990,55	990,54	IER3IP1	Immediate early response 3-interacting protein 1
	990,54	COL11A2	Isoform 1 of Collagen alpha-2(XI) chain
	990,56	CAPS	Calcyphosin
	990,56	SARS1	Seryl-tRNA synthetase
724,50	724,39	FLNA	Isoform 2 of Filamin-A
1064,60	1064,60	PDCD6IP	Programmed cell death 6-interacting protein
	1064,60	EPPK1	Epiplakin 1
	1064,60	RPL23A	60S ribosomal protein L23a
	1064,60	ALDH1A1	Retinal dehydrogenase 1
892,5	892,50	COL1A2	Collagen alpha-2(I) chain
	892,51	FUBP3	Isoform 1 of Far upstream element-binding protein 3
1203,70	1203,70	TAGLN	Transgelin
921,50	921,50	CRYAB	Alpha-crystallin B chain
	921,50	PSMD12	26S proteasome non-ATPase regulatory subunit 12
	921,50	BLVRA	Biliverdin reductase A
	921,50	OXCT1	Succinyl-CoA:3-ketoacid-coenzyme A transferase 1, mitochondrial
1261,65	1261,65	PYGB	Glycogen phosphorylase, brain form
	1261,65	DUSP23	Dual specificity protein phosphatase 23
875,45	875,46	ALG1	Chitobiosyldiphosphodolichol beta-mannosyltransferase
856,55	856,55	d.n.f	UPF0553 protein C9orf64
	856,55	ETFB	Isoform 1 of Electron transfer flavoprotein subunit beta
1547,85	1547,85	IDE	Insulin-degrading enzyme
838,45	838,45	VCL	Isoform 1 of Vinculin
827,50	827,49	VPS35	Vacuolar protein sorting-associated protein 35
890,55	890,55	SOD3	Extracellular superoxide dismutase [Cu-Zn]
	890,55	RRAS	Ras-related protein R-Ras
	890,55	SPON1	Spondin-1
	890,55	ATP5PF	ATP synthase-coupling factor 6, mitochondrial
827,55	827,54	IQGAP1	Ras GTPase-activating-like protein
	827,54	LRPPRC	Leucine-rich PPR motif-containing protein, mitochondrial
	827,54	SAE1	SUMO-activating enzyme subunit 1
	827,54	SMC1A	Structural maintenance of chromosomes protein 1A
	827,54	UBA1	Isoform 1 of Ubiquitin-like modifier-activating enzyme 6
	827,55	RPL8	60S ribosomal protein L8
731,45	731,45	COPG1	Coatomer subunit gamma 1
	731,45	RPL30	60S ribosomal protein L30

723,40	723,45	ATP5F1A	ATP synthase subunit alpha, mitochondrial
	723,48	CPT1A	Isoform 1 of Carnitine O-palmitoyltransferase 1, liver isoform

d.n.f: data not found

Table S6. Protein inference after *m/z* filtering from heatmap hippocampus 12m

Mass (*m/z*)	Near Mass Match	Gene name	Protein name
1340,70	1340,70	XPO1	Exportin-1
1340,72	1340,71	MYLK	Isoform 2 of Myosin light chain kinase, smooth muscle
	1340,73	MYH1	Myosin-1
1339,7	1339,70	FAM120A	Isoform F of Constitutive coactivator of PPAR-gamma-like protein 1
	1339,70	LRBA	Isoform 2 of Lipopolysaccharide-responsive and beige-like anchor protein
1339,74	1339,73	FKBP4	FK506-binding protein 4
	1339,76	DDX42	Isoform 1 of ATP-dependent RNA helicase DDX42
748,40	748,42	d.n.f	IPI00645488-R
	748,42	d.n.f	IPI00645488-R
1340,74	1340,74	MPO	Isoform H17 of Myeloperoxidase
	1340,74	MRPL4	Isoform 1 of 39S ribosomal protein L4, mitochondrial

d.n.f: data not found

Table S7. Protein inference after *m/z* filtering from top25 hippocampus 12m

Mass (*m/z*)	Near Mass Match	Gene name	Protein name
748,42	748,42	d.n.f	IPI00645488-R
1339,72	1339,72	P4HA2	Isoform IIa of Prolyl 4-hydroxylase subunit alpha-2
	1339,72	ETFB	Isoform 1 of Electron transfer flavoprotein subunit beta
	1339,72	FASN	Fatty acid synthase
1153,62	1153,62	ACADM	Isoform 1 of Medium-chain specific acyl-CoA dehydrogenase, mitochondrial
	1153,62	MRC1	Macrophage mannose receptor 1
1339,74	1339,73	ITGA5	Integrin alpha-5
	1339,73	FKBP4	FK506-binding protein 4
1340,74	1340,74	MPO	Isoform H17 of Myeloperoxidase
	1340,74	MRPL4	Isoform 1 of 39S ribosomal protein L4, mitochondrial
1154,62	1154,62	LMNB1	Lamin-B1
	1154,62	SYNM	Isoform 1 of Synemin
1339,70	1339,70	FAM120A	Isoform F of Constitutive coactivator of PPAR-gamma-like protein 1
	1339,70	LRBA	Isoform 2 of Lipopolysaccharide-responsive and beige-like anchor protein
729,40	729,41	HBA1	Hemoglobin subunit alpha
748,40	748,42	d.n.f	IPI00645488-R
881,52	881,52	MVP	Major vault protein
1153,60	1153,60	RPL34	60S ribosomal protein L34
	1153,60	OXA1L	Isoform 1 of Mitochondrial inner membrane protein
	1153,60	COPB1	Coatomer subunit beta
	1153,60	FTH1	Ferritin heavy chain
	1153,60	CHMP4B	Charged multivesicular body protein 4b
728,42	728,43	COL6A3	Isoform 1 of Collagen alpha-3(VI) chain
1154,60	1154,60	CAT	Catalase
	1154,60	PLS3	Plastin-3
	1154,60	PLS2	Plastin-2
1153,64	1153,63	THBS2	Thrombospondin-2
	1153,63	COL6A3	Isoform 1 of Collagen alpha-3(VI) chain
	1153,65	LRRC15	Leucine-rich repeat-containing protein 15
874,48	874,49	AHCY	Adenosylhomocysteinase
	874,49	FABP3	Fatty acid-binding protein, heart
1340,76	1340,78	H1-5	Histone H1.5
	1340,78	CFL1	Cofilin-1
	1340,78	CAMK2D	Isoform Delta 2 of Calcium/calmodulin-dependent protein kinase type II delta chain
	1340,78	CAP1	Adenylyl cyclase-associated protein
760,42	760,40	HP	Haptoglobin
801,48	801,48	ECH1	Delta(3,5)-Delta(2,4)-dienoyl-CoA isomerase, mitochondrial
	801,48	LAMC1	Laminin subunit gamma-1
	801,48	ALDOA	Fructose-bisphosphate aldolase A
1339,68	1339,68	OXA1L	Isoform 1 of Mitochondrial inner membrane protein
	1339,68	PYGB	Glycogen phosphorylase, brain form
760,40	760,40	HP	Haptoglobin
1153,58	1153,58	d.n.f	Isoform Long of ES1 protein homolog, mitochondrial
1144,62	1144,62	CFL1	Cofilin-1
	1144,62	ITIH4	Isoform 2 of Inter-alpha-trypsin inhibitor heavy chain H4
	1144,62	PTER	Isoform 1 of Phosphotriesterase-related protein
	1144,62	PPP2R5A	Serine/threonine-protein phosphatase 2A 56 kDa regulatory subunit alpha isoform
833,44	833,45	FHL1	Isoform 1 of Four and a half LIM domains protein 1
944,58	944,57	GRHPR	Glyoxylate reductase/hydroxypyruvate reductase
	944,58	GYG1	Isoform GN-1L of Glycogenin-1
	944,58	ACAD9	Acyl-CoA dehydrogenase family member 9, mitochondrial
	944,58	WFS1	Wolframin
	944,59	HNRNPH2	Heterogeneous nuclear ribonucleoprotein G
870,58	870,58	d.n.f	IPI00430411-R

d.n.f: data not found

Table S8. Protein inference after *m/z* filtering from heatmap cortex 12m

Mass (*m/z*)	Near Mass Match	Gene name	Protein name
862,46	862,47	DDX1	ATP-dependent RNA helicase DDX1
	862,47	HADHB	Trifunctional enzyme subunit beta, mitochondrial
	862,48	RABEP1	Isoform 1 of Rab GTPase-binding effector protein 1
	862,48	LGALS3	Galectin-3
	862,48	GOT1	Aspartate aminotransferase, cytoplasmic
	862,48	CDK5	Cell division protein kinase 5
	862,48	d.n.f	Putative uncharacterized protein RENBP
1204,62	1204,62	COL6A3	COL6A3 protein
	1204,62	VCL	Isoform 1 of Vinculin
	1204,62	CKM	Creatine kinase M-type
	1204,62	FH	Isoform Mitochondrial of Fumarate hydratase, mitochondrial
881,50	881,50	RPL15	60S ribosomal protein L15
	881,49	DMBT1	Isoform 1 of Deleted in malignant brain tumors 1 protein
760,38	760,40	HP	Haptoglobin
1165,56	1165,56	LRPPRC	Leucine-rich PPR motif-containing protein, mitochondrial
863,46	863,46	ECHS1	Enoyl-CoA hydratase, mitochondrial
	863,46	NUP93	Nuclear pore complex protein Nup93
1298,64	1298,64	ECH1	Delta(3,5)-Delta(2,4)-dienoyl-CoA isomerase, mitochondrial
	1298,65	PKP2	Isoform 1 of Plakophilin-2
	1298,63	KRT5	Keratin, type II cytoskeletal 5
839,44	839,44	SMDT1	UPF0466 protein C22orf32, mitochondrial
	839,44	MMP9	Matrix metalloproteinase-9
	839,44	SMDT1	UPF0466 protein C22orf32, mitochondrial
809,42	809,42	ANXA1	Annexin A1
	809,42	UQCRC1	Cytochrome b-c1 complex subunit 1, mitochondrial
	809,42	MUC6	Mucin 6, gastric
	809,43	FLNA	Isoform 2 of Filamin-A
792,38	792,36	ACTN1	Actinin, alpha 1 isoform a
	792,40	NEXN	Isoform 1 of Nexilin
966,48	966,48	d.n.f	cDNA FLJ51518, highly similar to Annexin A11
	966,48	EPRS	Bifunctional aminoacyl-tRNA synthetase
	966,49	NCBP1	Nuclear cap-binding protein subunit 1
	966,49	RPL10A	60S ribosomal protein L10a
761,40	761,40	MYH2	Myosin-2
	761,42	PLIN3	Isoform B of Perilipin-3
	761,42	AIFM1	Isoform 1 of Apoptosis-inducing factor 1, mitochondrial
1205,62	1205,62	LAMB2	Laminin subunit beta-2
	1205,62	FAM83H	Protein FAM83H
775,42	775,42	RAB8B	Ras-related protein Rab-8B
	775,42	RAB1A	Isoform 1 of Ras-related protein Rab-1A
	775,42	RPS18	40S ribosomal protein S18
938,48	938,48	EHD4	EH domain-containing protein 4
	938,48	CLTC	Isoform 1 of Clathrin heavy chain 1
	938,48	GC	Isoform 1 of Vitamin D-binding protein
890,46	890,46	CLTC	Isoform 1 of Clathrin heavy chain 1
	890,46	KRT18	Keratin, type I cytoskeletal 18
	890,46	HSPB7	Isoform 2 of Heat shock protein beta-7
	890,48	HMGCL	Hydroxymethylglutaryl-CoA lyase, mitochondrial
762,40	762,41	KRT19	Keratin, type I cytoskeletal 19
	762,41	EEF1G	Elongation factor 1-gamma
	762,39	TGFB1I1	Isoform 2 of Transforming growth factor beta-1-induced transcript 1 protein
994,50	994,50	d.n.f	G1 to S phase transition 1 isoform 2
	994,50	MYH10	Isoform 1 of Myosin-10
	994,50	RPL6	60S ribosomal protein L6
	994,50	DCXR	L-xylulose reductase
	994,51	GLUL	Glutamine synthetase
893,48	893,48	d.n.f	Isoform 1 of Uncharacterized protein C9orf142
923,50	923,49	NDUFA7	NADH dehydrogenase [ubiquinone] 1 alpha subcomplex subunit 7
	923,50	AKR1C1	Aldo-keto reductase family 1 member C1
	923,52	SLC25A5	ADP/ATP translocase 2
808,38	808,38	ENO1	Isoform alpha-enolase of Alpha-enolase
1166,58	1166,58	SULT1A1	Sulfotransferase 1A2
	1166,58	d.n.f	Phenol sulfotransferase 1A5*1A possible alternative splicing form
	1166,58	APCS	Serum amyloid P-component

1135,58	1135,58	SPTAN1	Isoform 1 of Spectrin alpha chain, brain
	1135,58	FKBP4	FK506-binding protein 4
1165,58	1165,58	KRT10	Keratin, type I cytoskeletal 10
	1165,58	TXN	Thioredoxin
	1165,58	PMVK	Phosphomevalonate kinase
	1165,58	d.n.f	cDNA, FLJ93744, highly similar to Homo sapiens keratin 6E (KRT6E), mRNA
	1165,58	PMVK	Phosphomevalonate kinase
	1165,58	DSP	Isoform DPI of Desmoplakin
805,42	805,42	TSPAN8	Tetraspanin-8
	805,42	FKBP10	FK506-binding protein 10
1354,62	1354,62	d.n.f	cDNA FLJ59211, highly similar to Glucosidase 2 subunit beta
	1354,62	CPT2	Carnitine O-palmitoyltransferase 2, mitochondrial
	1354,62	ACTB	Actin, cytoplasmic 1
	1354,62	VAT1	Synaptic vesicle membrane protein VAT-1 homolog
1355,64	1355,64	d.n.f	59 kDa protein
	1355,64	ACSL3	Long-chain-fatty-acid--CoA ligase 3
1354,64	1354,64	CA2	Carbonic anhydrase 2
	1354,64	PALLD	Isoform 4 of Palladin
	1354,64	RAB4A	RAB4A, member RAS oncogene family variant
1392,74	1392,74	KRT6A	Keratin, type II cytoskeletal 6A
1391,74	1391,74	CCT5	T-complex protein 1 subunit epsilon
	1391,74	GARS	Glycyl-tRNA synthetase
1295,60	1295,61	TAGLN	Transgelin
	1295,59	TTN	Titin isoform N2-A
1391,72	1391,72	ATP2A1	Isoform SERCA1B of Sarcoplasmic/endoplasmic reticulum calcium ATPase 1
	1391,72	EIF3A	Eukaryotic translation initiation factor 3 subunit A
1392,72	1392,72	CRP	Isoform 1 of C-reactive protein
	1392,72	TALDO1	Transaldolase
	1392,72	COL6A1	Collagen alpha-1(VI) chain

d.n.f: data not found

Table S9. Protein inference after *m/z* filtering from top25 cortex 12m

**Mass (*m/z*)**	**Near Mass Match**	**Gene name**	**Protein name**
809,44	809,44	EEF1A2	Elongation factor 1-alpha 2
	809,44	DSP	Isoform DPI of Desmoplakin
	809,44	KRT19	Keratin, type I cytoskeletal 19
	809,44	KRT9	Keratin, type I cytoskeletal 9
	809,44	KRT10	Keratin, type I cytoskeletal 10
	809,44	KRT17	Keratin, type I cytoskeletal 17
	809,44	KRT14	Keratin, type I cytoskeletal 14
	809,44	KRT16	Keratin, type I cytoskeletal 16
	809,44	TRA2B	Isoform 1 of Transformer-2 protein homolog beta
	809,44	KRT15	Keratin, type I cytoskeletal 15
862,46	862,45	TNNI1	Troponin I, slow skeletal muscle
	862,47	DDX1	ATP-dependent RNA helicase
	862,47	HADHB	Trifunctional enzyme subunit beta, mitochondrial
1391,74	1391,74	CCT5	T-complex protein 1 subunit epsilon
	1391,74	GARS1	Glycyl-tRNA synthetase
1391,76	1391,76	PGD	6-phosphogluconate dehydrogenase, decarboxylating
850,58	850,53	H2AC18	Histone H2A type 2-A
	850,53	H2AC1	Histone H2A type 1-A
	850,53	H2AZ2	Histone H2A.V
	850,53	H2AC6	Histone H2A type 1-C
959,58	959,59	TP53I3	Isoform 1 of Quinone oxidoreductase PIG3
	959,59	H3Y1	Histone H3.1
	959,59	d.n.f	18 kDa protein
881,50	881,50	RPL15	60S ribosomal protein L15
	881,52	MVP	Major vault protein
1391,72	1391,72	ATP2A2	Isoform SERCA2B of Sarcoplasmic/endoplasmic reticulum calcium ATPase 2
	1391,72	ATP2A1	Isoform SERCA1B of Sarcoplasmic/endoplasmic reticulum calcium ATPase 1
	1391,72	ATP2A3	Isoform SERCA3B of Sarcoplasmic/endoplasmic reticulum calcium ATPase 3
	1391,72	EIF2A	Eukaryotic translation initiation factor 3 subunit A
1295,62	1295,62	SMC1A	Structural maintenance of chromosomes protein 1A
	1295,62	C11orf54	Isoform 1 of Ester hydrolase C11orf54
841,48	841,48	USP27X	Ubiquitin carboxyl-terminal hydrolase 14
	841,49	CLCA1	Calcium-activated chloride channel regulator 1
1354,64	1354,64	CA2	Carbonic anhydrase 2
	1354,64	PALLD	Isoform 4 of Palladin
	1354,64	RAB4A	Ras-related protein Rab-4A
743,46	743,47	AP2B1	Isoform B of AP-2 complex subunit alpha-1
	743,47	AP2A1	Isoform 2 of AP-2 complex subunit alpha-2
1295,62	1295,61	TAGLN	Transgelin
760,36	760,40	HP	Haptoglobin
1202,64	1202,64	TALDO1	Transaldolase
1355,64	1355,64	d.n.f	59 kDa protein
	1355,64	SLC27A3	Long-chain-fatty-acid--CoA ligase 3
862,44	862,45	TNNI1	Troponin I, slow skeletal muscle
1355,66	1355,66	PPP1R12C	Isoform 1 of Protein phosphatase 1 regulatory subunit 12C
	1355,66	TUBB2A	Tubulin beta-2A chain
	1355,66	TTN	Titin isoform N2-A
743,44	743,44	TPSAB1	Na+/K+ -ATPase alpha 1 subunit isoform c
	743,44	G6PD	Isoform Long of Glucose-6-phosphate 1-dehydrogenase
	743,44	LRBA	Isoform 2 of Lipopolysaccharide-responsive and beige-like anchor protein
	743,44	GMPR2	GMP reductase 2
	743,44	ITGB7	Integrin beta-2
840,48	840,49	SF3B3	Isoform 1 of Splicing factor 3B subunit 3
1127,56	1127,56	VPS35	Vacuolar protein sorting-associated protein 35
731,40	731,40	d.n.f	cDNA FLJ56425, highly similar to Very-long-chain specific acyl-CoAdehydrogenase, mitochondrial
	731,40	GPI	Glucose-6-phosphate isomerase
	731,40	d.n.f	Gelsolin-like capping protein
	731,40	FH	Isoform Mitochondrial of Fumarate hydratase, mitochondrial
	731,40	d.n.f	Uncharacterized protein C6orf203
833,42	833,42	VCL	Isoform 1 of Vinculin
808,38	808,38	ENO1	Isoform alpha-enolase of Alpha-enolase
1296,62	1296,61	PLEC	Isoform 4 of Plectin-1
	1296,61	UBE2L3	Ubiquitin-conjugating enzyme E2 L3
	1296,61	SPTBN1	Isoform Long of Spectrin beta chain, brain 1
	1296,63	PCCA	Propionyl-Coenzyme A carboxylase, alpha polypeptide isoform a precursor
	1296,63	DDX39B	Isoform 1 of Spliceosome RNA helicase
	1296,63	d.n.f	cDNA FLJ55484, highly similar to ATP-dependent RNA helicase DDX39

d.n.f: data not found

Table S10. List of antibodies used in this study.

Antibody	Host animal	Work dilution	Reference (Cat #)	Company	Secondary antibody
ACAD9	Rabbit / IgG polyclonal	1:50-1:100	PA5-76270	Thermo Fisher Scientific	1:200 Alexa Fluor® 488 donkey anti-rabbit. Invitrogen (Massachusetts, USA)
ACSL3	Mouse / IgG monoclonal	1:50-1:500	sc-166374	Santa Cruz Biotechnology	1:200 Alexa Fluor® 488 chicken anti-mouse. Invitrogen (Massachusetts, USA)
COL6A3	Rabbit / IgG polyclonal	1:100-1:200	ab231025	Abcam	1:200 Alexa Fluor® 488 donkey anti-rabbit. Invitrogen (Massachusetts, USA)
Thioredoxin 1	Rabbit / IgG polyclonal	1:100-1:200	2429	Cell Signaling Technology	1:200 Alexa Fluor® 488 donkey anti-rabbit. Invitrogen (Massachusetts, USA)
Catalase	Mouse / IgG monoclonal	1:50-1:500	sc-271803	Santa Cruz Biotechnology	1:200 Alexa Fluor® 488 chicken anti-mouse. Invitrogen (Massachusetts, USA)
CA II	Mouse / IgG monoclonal	1:50-1:500	sc-48351	Santa Cruz Biotechnology	1:200 Alexa Fluor® 488 chicken anti-mouse. Invitrogen (Massachusetts, USA)
COPG	Mouse / IgG monoclonal	1:50-1:500	sc-271362	Santa Cruz Biotechnology	1:200 Alexa Fluor® 488 chicken anti-mouse. Invitrogen (Massachusetts, USA)
MPO light chain	Mouse / IgG monoclonal	1:50-1:500	sc-390109	Santa Cruz Biotechnology	1:200 Alexa Fluor® 488 chicken anti-mouse. Invitrogen (Massachusetts, USA)
Prohibitin 2	Mouse / IgG monoclonal	1:50-1:500	sc-133094	Santa Cruz Biotechnology	1:200 Alexa Fluor® 488 chicken anti-mouse. Invitrogen (Massachusetts, USA)
TCP-1 ε	Mouse / IgG monoclonal	1:50-1:500	sc-376188	Santa Cruz Biotechnology	1:200 Alexa Fluor® 488 chicken anti-mouse. Invitrogen (Massachusetts, USA)
Transgelin	Mouse / IgG monoclonal	1:50-1:500	sc-53932	Santa Cruz Biotechnology	1:200 Alexa Fluor® 488 chicken anti-mouse. Invitrogen (Massachusetts, USA)
Vinculin	Mouse / IgG monoclonal	1:100-1:200	14977782	Thermo Fisher Scientific	1:200 Alexa Fluor® 488 chicken anti-mouse. Invitrogen (Massachusetts, USA)
PDI	Mouse / IgG monoclonal	1:50-1:500	sc-74551	Santa Cruz Biotechnology	1:200 Alexa Fluor® 488 chicken anti-mouse. Invitrogen (Massachusetts, USA)
PRDX1	Rabbit / IgG polyclonal	1:200-1:250	PA3-750	Thermo Fisher Scientific	1:200 Alexa Fluor® 488 donkey anti-rabbit. Invitrogen (Massachusetts, USA)
HADHA	Rabbit / IgG polyclonal	1:100-1:1000	SAB2700479	Merck	1:200 Alexa Fluor® 488 donkey anti-rabbit. Invitrogen (Massachusetts, USA)
HSPA8	Rabbit / IgG polyclonal	1:100-1:1000	SAB2701964	Merck	1:200 Alexa Fluor® 488 donkey anti-rabbit. Invitrogen (Massachusetts, USA)
RPL23	Rabbit / IgG polyclonal	1:50-1:200	NBP1-87847	Novus Biologicals	1:200 Alexa Fluor® 488 donkey anti-rabbit. Invitrogen (Massachusetts, USA)
RPL13A	Rabbit / IgG polyclonal	1:50-1:200	NBP1-92345	Novus Biologicals	1:200 Alexa Fluor® 488 donkey anti-rabbit. Invitrogen (Massachusetts, USA)
RPL30	Rabbit / IgG polyclonal	1:50-1:100	PA5-89360	Thermo Fisher Scientific	1:200 Alexa Fluor® 488 donkey anti-rabbit. Invitrogen (Massachusetts, USA)
RPL17	Rabbit / IgG polyclonal	1:50-1:200	SAB4500019	Thermo Fisher Scientific	1:200 Alexa Fluor® 488 donkey anti-rabbit. Invitrogen (Massachusetts, USA)
RPL15	Rabbit / IgG polyclonal	1:100-1:500	PA5-106578	Thermo Fisher Scientific	1:200 Alexa Fluor® 488 donkey anti-rabbit. Invitrogen (Massachusetts, USA)
PPP2R5A	Rabbit / IgG polyclonal	1:100-1:500	PA5-106739	Thermo Fisher Scientific	1:200 Alexa Fluor® 488 donkey anti-rabbit. Invitrogen (Massachusetts, USA)
DYNC1H1	Rabbit / IgG polyclonal	1:100-1:500	PA5-115149	Thermo Fisher Scientific	1:200 Alexa Fluor® 488 donkey anti-rabbit. Invitrogen (Massachusetts, USA)
Exportin-1/CRM1	Rabbit / IgG polyclonal	1:200-1:800	46249	Cell Signaling Technology	1:200 Alexa Fluor® 488 donkey anti-rabbit. Invitrogen (Massachusetts, USA)
eIF3a	Rabbit / IgG polyclonal	1:100-1:500	PA5-17212	Thermo Fisher Scientific	1:200 Alexa Fluor® 488 donkey anti-rabbit. Invitrogen (Massachusetts, USA)

Table S11. Sequence alignment analysis by BLAST.

	m/z MALDI	Protein name	Human sequence	Mouse sequence	Query Cover	E value	% identity
Hippocampus 6m	920,50	PRDX1	GLFIIDDK	GLFIIDDK	1,00	0,05	1
	955,57	P4HB	ILEFFGLK	ILEFFGLK	1,00	0,05	1
	1039,56	DYNC1H1	TMTLFSALR	TMTLFSALR	1,00	0,01	1
	1040,55	HADHA	FVDLYGAQK	FVDLYGAQK	1,00	0,01	1
	1109,60	COPG1	VVLEHEEVR	VVLEHEEVR	1,00	0,00	1
	1154,60	CAT	LCENIAGHLK	LCENIAGHLK	1,00	0,00	1
	1154,60	COL6A3	SSIMAFAIGNK	SIMALAVGSK	0,90	5,80	0,7
Cortex 6m	838,45	VCL	GLVAEGHR	GLVAEGHR	1	0,18	1
	858,45	HSPA8	GTLDPVEK	GTLDPVEK	1,00	0,13	1
	1064,60	RPL23A	KLYDIDVAK	KLYDIDVAK	1,00	0,01	1
	1202,65	PHB2	DLQMVNISLR	DLQMVNISLR	1,00	0,00	1
	1355,65	ACSL3	NTPLCDSFVFR	TPLCDRFVFR	0,90	0,00	0,9
	1204,64	RPL17	SAEFLLHMLK	SAEFLLHMLK	1,00	0,00	1
Hippocampus 12m	1340,7	XPO1	SAFPHLQDAQVK	SAFPHLQDAQVK	1	0,000006	1
	1340,74	MPO	IGLDLPALNMQR	IGLDLPALNMQR	1,00	0,00	1
	1144,62	PPP2R5A	DTTLTEPVIR	DTTLTEPVIR	1,00	0,00	1
	944,58	ACAD9	TVETLLLR	TVETLLLR	1,00	0,13	1
Cortex 12m	1354,64	CAII	GKSADFTNFDPR	GKRAAFANFDP	0,91	1,2	0,7273
	1165,58	TXN	VGEFSGANKEK	VGEFSGANKEK	1,00	0,00	1
	1391,72	EIF3A	QPALDVLYDVMK	QPALDVLYDVMK	1,00	0,00	1
	881,50	RPL15	NTLQLHR	NTLQLHR	1,00	0,43	1
	1204,32	VCL	DIAKASDEVTR	DIAKASDEVTR	1,00	0,00	1
	1295,60	TAGLN	EFTESQLQEGK	DFTDSQLQEGK	1,00	0,00	0,8182

A table view with one row per hit, showing the accession number with BLAST output scores.
Query Coverage represents the percent of the query length that is included in the aligned segments.
E[xpect] Value represents the number of alignments expected by chance with the calculated score or better. The expect value is the default sorting metric; for significant alignments, the E value should be very close to zero.
% Identity represents the highest percent identity for a set of aligned segments to the same subject sequence.

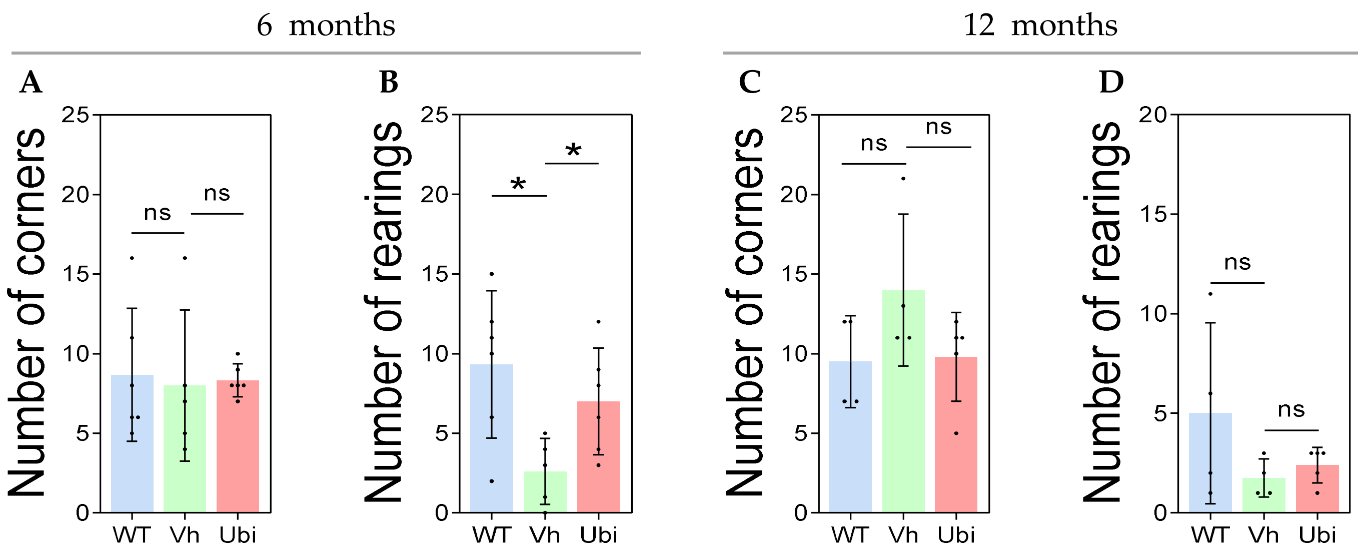

Figure S1. (A,C) Exploratory activity of mice in the neophobia test. Each column represents the average number of corners visited by each experimental group (n = 6) after 6 (**A**) or 12 (**C**) months of age. Values are expressed as mean ± S.E.M. * = $p < 0.05$. (**B,D**) Anxiety levels of the mice studied by the open-field test. Each column represents the average number of rearings performed by each mouse according to its experimental group (n= 6), at after 6 (**B**) or 12 (**D**) months of age. Values are expressed as mean ± S.E.M. * = $p < 0.05$.

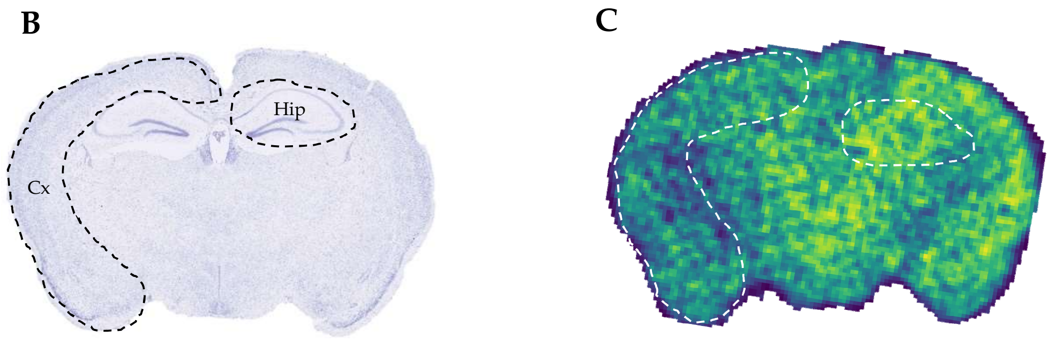

Figure S2. (A) Representative Nissl-stained mouse brain tissue section of a 6-m old Vh animal (−1,82 mm from bregma). Annotations: Cx, cortex; Hip, hippocampal region. Each studied region is delineated with a black dashed line. (**B**) Molecular image of peptide ion detected at m/z 733,2. Each studied region is delineated with a white dashed line.

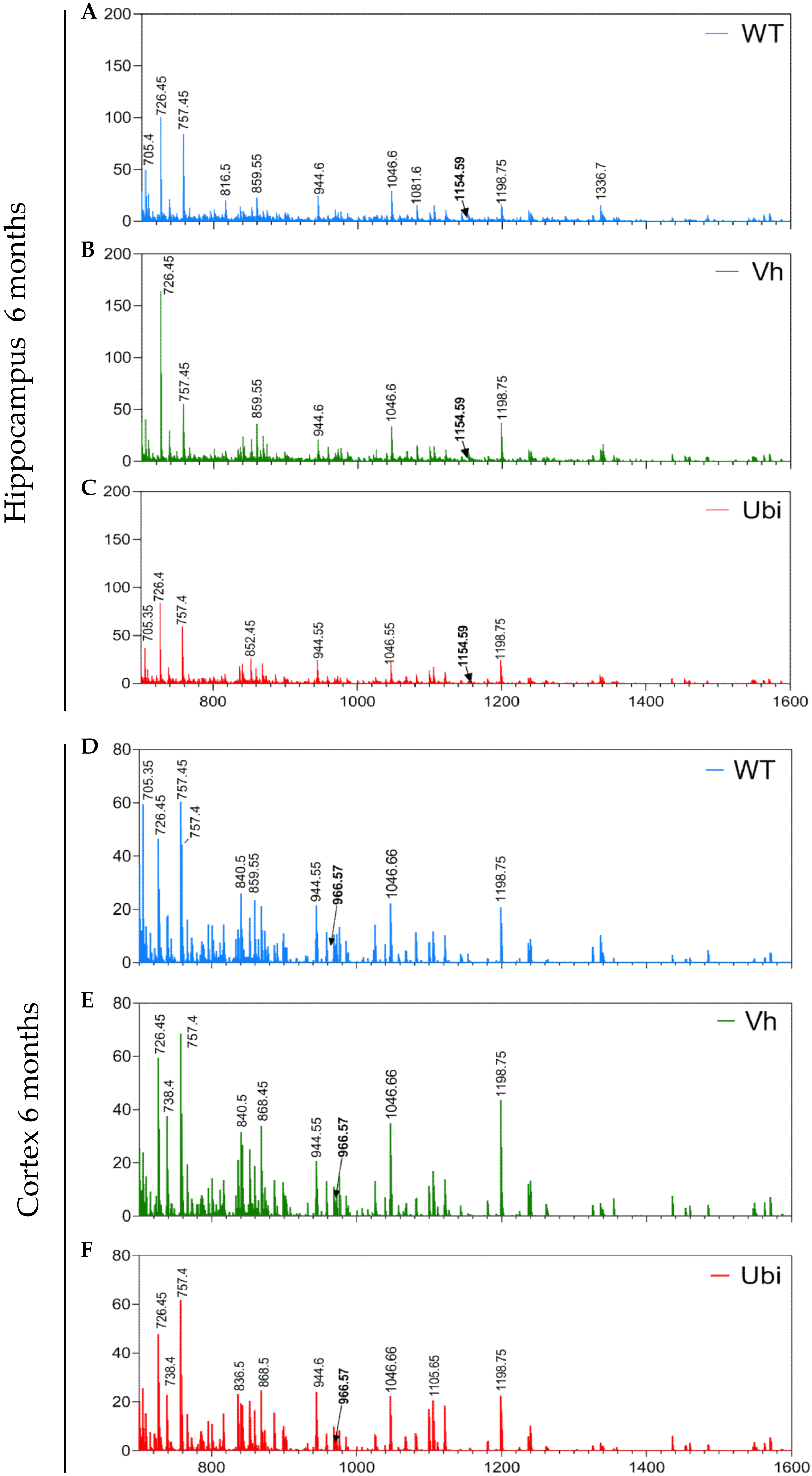

Figure S3. Comparison of mass spectra obtained from MALDI-MSI, identified from hippocampus and cortex at 6 months of age within the intact peptide mass range (m/z 700–1600). (**A-C**) Mass spectrum of hippocampus for WT, Vh and Ubi conditions, respectively. (**D-F**) Mass spectrum of cortex for WT, Vh and Ubi conditions, respectively. To facilitate comparison, WT spectra are colored in blue, Vh in green and Ubi in red. All spectra presented here are in positive ion mode.

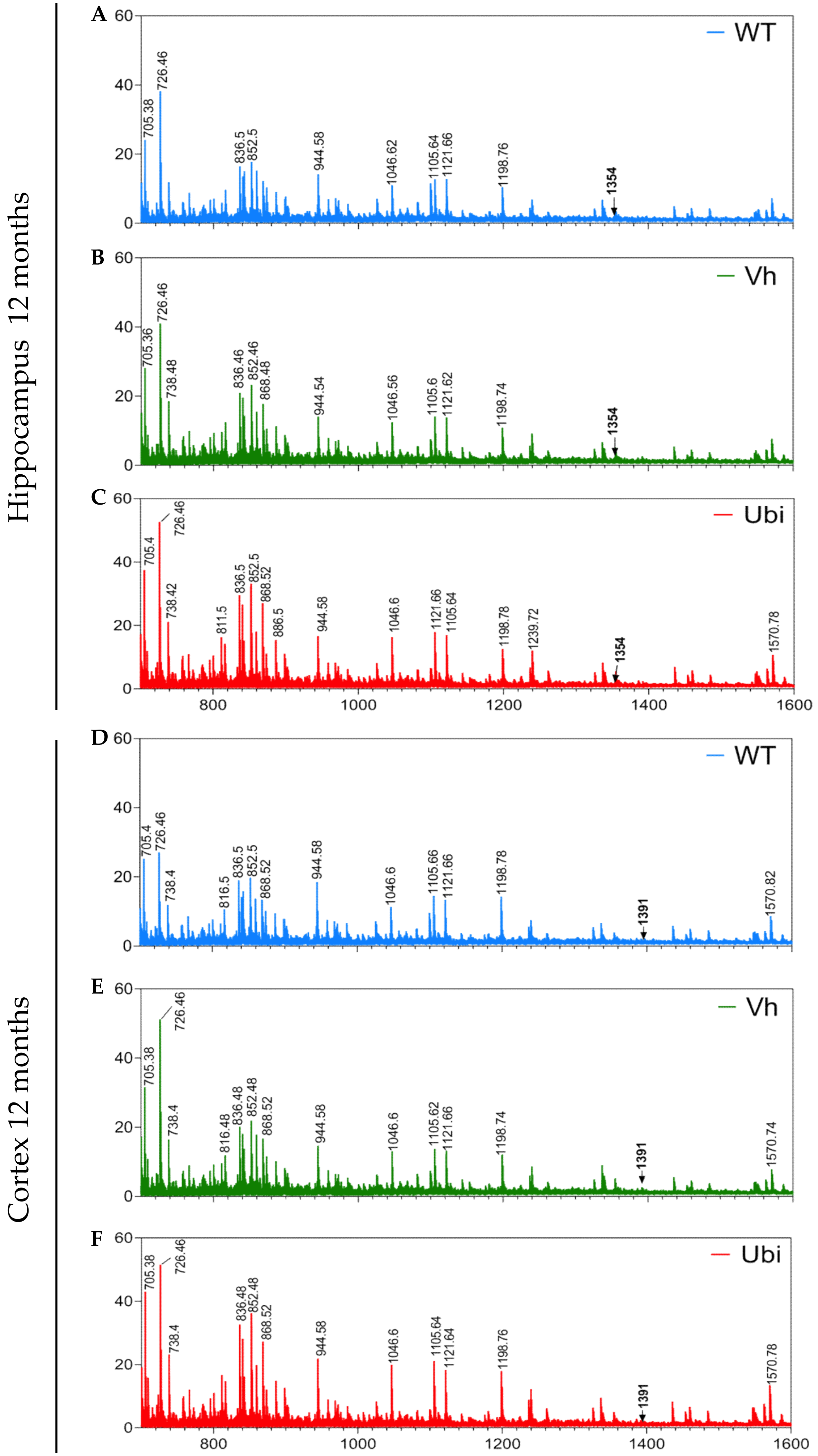

Figure S4. Comparison of mass spectra obtained from MALDI-MSI, identified from hippocampus and cortex at 12 months of age within the intact peptide mass range (m/z 700–1600). (**A-C**) Mass spectrum of hippocampus for WT, Vh and Ubi conditions, respectively. (**D-F**) Mass spectrum of cortex for WT, Vh and Ubi conditions, respectively. To facilitate comparison, WT spectra are colored in blue, Vh in green and Ubi in red. All spectra presented here are in positive ion mode.

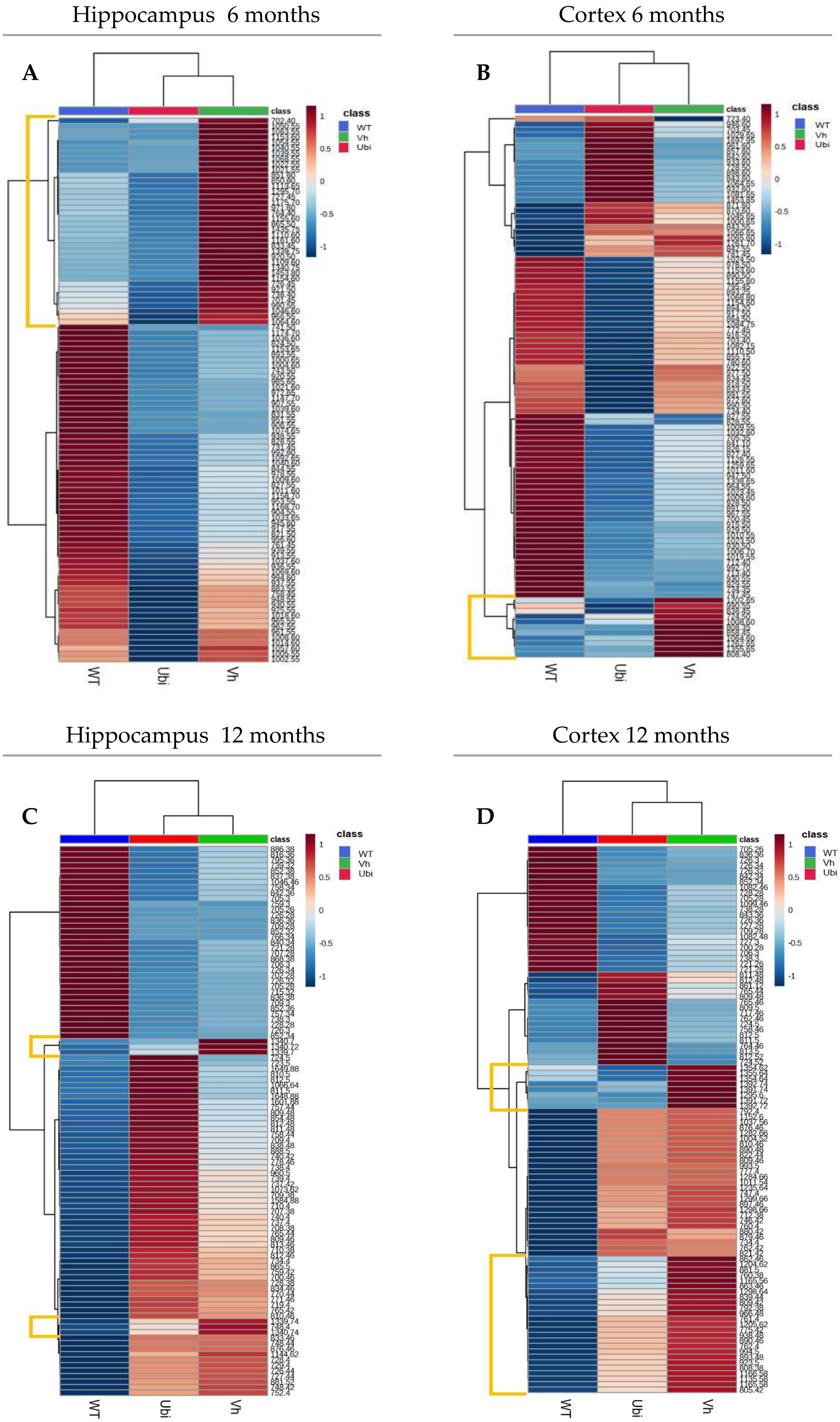

Figure S5. Clustered heatmap of the hippocampal 6m (**A**), cortical 6m (**B**), hippocampal 12m (**C**), cortical 12m (**D**) region performed in MetaboAnalyst 3.0 using intensity data. Each heatmap represents the 100 most significant masses among the experimental conditions and each row represent a mass. Hierarchical ranking was performed using Pearson's correlation coefficient. Relative expression (arbitrary units) is shown in red for high values and in dark blue for low values. Orange brackets refer to those masses selected for further identification to proteins.

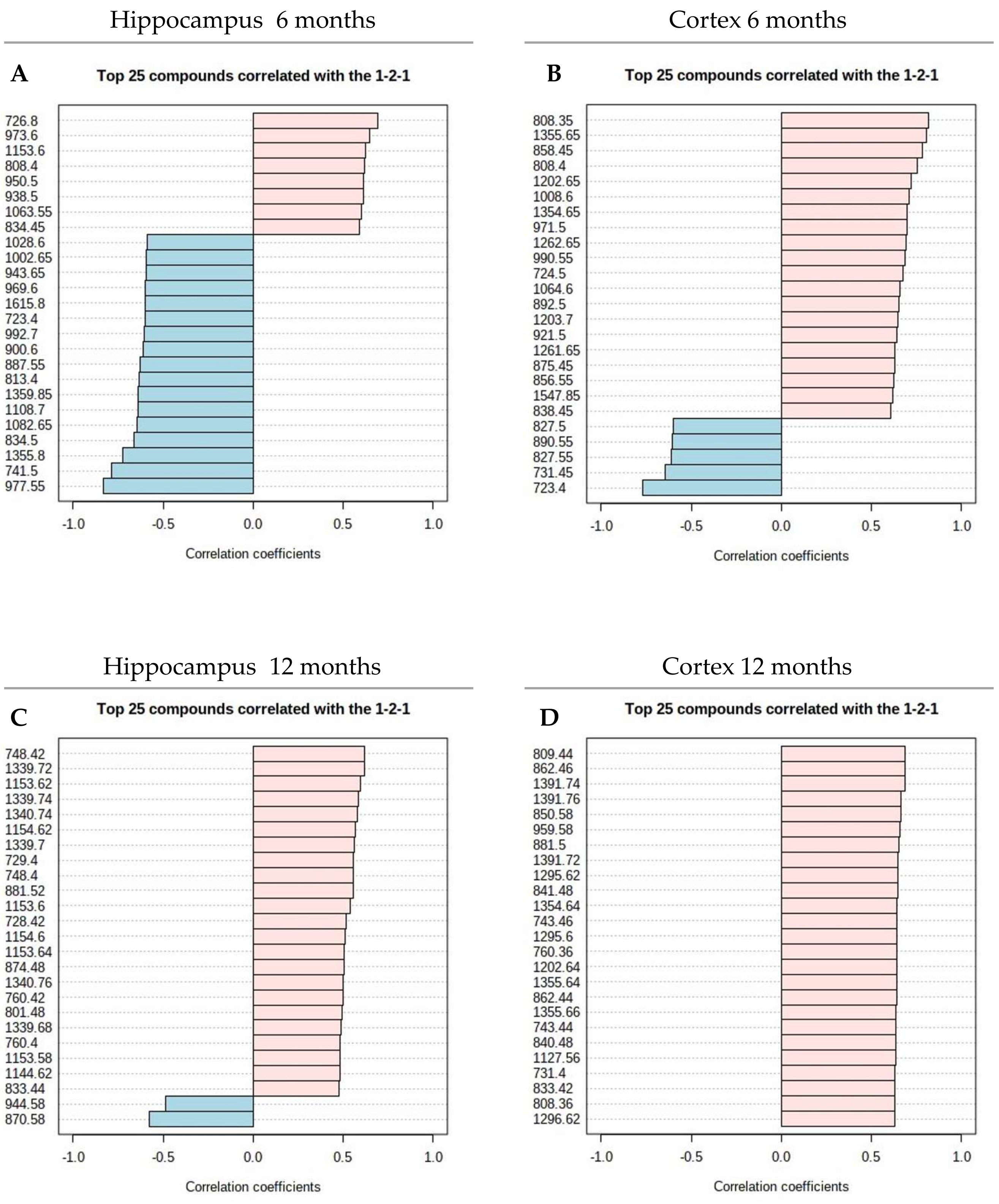

Figure S6. Top 25 correlations with the normalized intensity of hippocampal 6m (**A**), cortical 6m (**B**), hippocampal 12m (**C**), cortical 12m (**D**) area. Correlation analysis identified peptides associated with '1–2–1' pattern (WT vs Vh and Ubi vs Vh). The peptides are represented as horizontal bars, with colors in light pink indicating positive correlations and that in light blue indicating negative correlations.
